# Altered Molecular Composition of a Specific Subset of Prefrontal Cortical Excitatory Synapses in Schizophrenia

**DOI:** 10.1523/JNEUROSCI.0645-25.2025

**Published:** 2025-08-19

**Authors:** Andrea Lorincz, Maria Ashaber, Zoltan Nusser

**Affiliations:** Laboratory of Cellular Neurophysiology, HUN-REN Institute of Experimental Medicine, Budapest 1083, Hungary

**Keywords:** excitatory synapses, human, interneuron, NMDA receptor, prefrontal cortex, schizophrenia

## Abstract

Abnormal excitatory synaptic transmission in the human prefrontal cortex has been implicated in the pathophysiology of schizophrenia based primarily on genetic evidence. However, changes in synaptic function cannot be predicted from altered gene expressions, but determining the amount, density, and subsynaptic distribution of synaptic proteins is the only reliable indirect readout of function. Detecting proteins in individual synapses of human postmortem tissues has been severely constrained by technical limitations. Here we overcome this limitation by optimizing a high-resolution, quantitative localization method to facilitate antigen recognition at excitatory synapses in postmortem brains of both sexes. Using PSD-95 immunoreactivity as molecular marker of excitatory synapses, we demonstrate the lack of significant differences in synapse density and size in upper cortical layers of control and schizophrenia subjects. The synaptic densities of postsynaptic AMPA and NMDA receptor subunits and presynaptic molecules Bassoon and Munc13-1 are also indistinguishable between control and schizophrenia subjects. The number of Munc13-1 nanoclusters, marking presynaptic neurotransmitter release sites, does not differ either. Excitatory synapses on parvalbumin expressing interneurons contain similar AMPA but significantly lower NMDA receptor densities in schizophrenia compared with control subjects. Our study provides the first comprehensive comparison of key functionally relevant synaptic proteins in individual human excitatory synapses and demonstrates that changes in the molecular composition of only a specific subset of excitatory synapses may contribute to the pathophysiology of schizophrenia.

## Significance Statement

Abnormal excitatory synaptic transmission in the prefrontal cortex has been implicated in the pathophysiology of schizophrenia. Our study provides novel insights into the molecular mechanisms underlying excitatory synaptic dysfunction in schizophrenia. By utilizing a high-resolution localization method with improved antigen recognition, we provide a comprehensive analysis of the density and subsynaptic distribution of key synaptic proteins in human cortical excitatory synapses. While we found no significant difference in overall synaptic densities and molecular compositions of excitatory synapses, our results reveal a reduction in NMDA receptor density in synapses targeting parvalbumin expressing interneurons in schizophrenia subjects. These findings suggest that changes in the molecular composition of only a specific subset of cortical synapses may contribute to the pathophysiology of schizophrenia.

## Introduction

Schizophrenia is a severe neuropsychiatric disorder affecting ∼1% of the population. While psychosis is a prominent clinical manifestation, the core features of schizophrenia encompass social impairments and cognitive decline. The dorsolateral prefrontal cortex (DLPFC) plays a central role in the pathophysiology of schizophrenia, particularly in deficits in working memory (Callicott et al., 2003; Cho et al., 2006; Lewis and Mirnics, 2006; Haenschel et al., 2009; Sigurdsson and Duvarci, 2015; Uhlhaas and Singer, 2015; Millan et al., 2016). Genetic (Kirov et al., 2012; Fromer et al., 2014; Purcell et al., 2014; Trubetskoy et al., 2022) and transcriptomic (Batiuk et al., 2022; Ruzicka et al., 2024) studies indicated that deficits in glutamatergic neurotransmission may contribute to cognitive impairments as they pointed to molecules enriched in glutamatergic synapses.

Synapses are highly complex structures built from thousands of proteins (Bayes and Grant, 2009). Extensive works revealed dozens of key pre- and postsynaptic proteins with pivotal roles in synaptic vesicle docking, priming, release, and postsynaptic response generation (Sudhof, 2012). For example, Bassoon is an essential component of the presynaptic active zone (AZ) and organizes synaptic vesicle pools via regulation of phosphorylation and cAMP homeostasis (Montenegro-Venegas et al., 2022). Munc13-1 is a key molecule in vesicle priming (Brose et al., 1995; Augustin et al., 1999), which forms small nanoclusters at the presynaptic AZ, the number of which correlates with the number of vesicle release sites (Reddy-Alla et al., 2017; Sakamoto et al., 2018; Karlocai et al., 2021). While mutations in Bassoon and Munc13-1 have been identified in patients with schizophrenia (Fromer et al., 2014; Chen et al., 2021) and other CNS disorders (Lipstein et al., 2017), the potential impact of changes in their densities and subsynaptic distribution in schizophrenia remains unclear. The quantal size of postsynaptic response is primarily determined by the density of AMPA-type glutamate receptors (AMPAR), whereas NMDA receptors (NMDAR) play a key role in long-term plasticity. The glutamate hypothesis (Krystal et al., 1994) postulates that malfunction of glutamate receptors contributes to the pathophysiology of schizophrenia. Accordingly, several studies have reported altered mRNA expression of AMPAR and NMDARs (Conti et al., 1994; Rubio et al., 2012) in various cortical areas of schizophrenia patients, sometimes with conflicting results.

However, the level of mRNA does not necessarily predict the amount of translated protein. Protein levels in postmortem tissues have been mainly assessed by Western blot analysis. Total homogenate of gray matter includes synaptic proteins that are not only associated with synapses but are located either intracellularly or extrasynaptically. Therefore, these studies cannot provide adequate information about the synaptic levels of proteins. While attempts have been made to compare protein levels isolated from postsynaptic density (PSD) fractions (Catts et al., 2015), these samples are pooled from diverse populations of synapses. Given the complexity in the molecular composition of cortical excitatory synapses, which underlie their functional diversity (Atwood and Karunanithi, 2002), it is crucial to investigate the level and subsynaptic distribution of synaptic molecules at individual synapses. High-resolution immunolocalization would be the obvious method of choice, but localization of synaptic proteins using pre-embedding immunolocalization in well-fixed thick tissue sections is prone to errors due to the inaccessibility of epitopes embedded into dense protein matrices of AZs and PSDs (Fritschy et al., 1998; Watanabe et al., 1998; Lorincz and Nusser, 2010; Konno et al., 2023). Successful synaptic detection therefore often requires very mild fixation and antigen retrieval. Mild fixation used for rodents is suboptimal for long-term tissue storage, which is the primary goal of pathologists when preserving the postmortem human tissue.

To address these limitations, here we adopted and further improved a multiplexed, high-resolution postembedding immunolocalization method (Holderith et al., 2020), allowing the localization of dozens of proteins within individual synapses in human DLPFC with a resolution of ∼40 nm. Here, we compared the size and density of excitatory synapses, as well as the amount and subsynaptic distribution of key pre- and postsynaptic proteins in schizophrenia and control subjects.

## Materials and Methods

### Human brain samples

Male and female human brain samples (Table 1) were obtained from the Department of Pathology, Szent Borbála Hospital via the Human Brain Research Laboratory (HUN-REN Institute of Experimental Medicine). All procedures were approved by the Regional Committee of Science and Research Ethics of Scientific Council of Health (ETT TUKEB 15032/2019/EKU, modified in 2023) and carried out in compliance with the Declaration of Helsinki. Control subjects had no history of psychiatric or neurologic deficits, and their deaths were not caused directly by any brain damage. Schizophrenia patients were diagnosed by ICD10-criteria (F20.x diagnoses). Relevant features of the subjects are summarized in Table 1. Brains were removed postmortem [postmortem time interval (PMI), 3.3 ± 0.7 h], and vertebral arteries and the internal carotid were cannulated. Perfusion was carried out using physiological saline containing 0.33% heparin (1.5 L for 30 min), followed by Zamboni fixative containing 4% paraformaldehyde, 0 or 0.05% glutaraldehyde, and ∼0.2% w/v picric acid in 0.1 M Na-phosphate buffer (PB), pH 7.4 (4 L for 2 h). Tissue blocks were removed and postfixed overnight in the same fixative without glutaraldehyde and then washed in 0.1 M PB, cryoprotected in 30% sucrose solution in 0.1 M PB at 4°C for 2 d before they were frozen over liquid nitrogen and stored at −80°C.

**Table 1.** Summary of control and schizophrenia subjects

Subject code	Condition	Gender	Age (years)	PMI (hh:mm)	Cause of death (ICD-10)
SKO2	Control	Male	74	4:55	Bronchopneumonia (J18.0)
SKO7	Control	Male	55	3:39	Pulmonary embolism with acute cor pulmonale (I25.0)
SKO9	Control	Female	78	3:45	Heart failure unspecified (I50.9)
SKO13	Control	Female	60	3:25	Respiratory arrest (R09.2)
SKO16	Control	Male	73	2:22	Respiratory arrest (R09.2)
SKO18	Control	Male	85	2:52	Congestive heart failure (I50.0)
SKO19	Control	Female	61	2:53	Acute transmural infarction of inferior wall (I21.1)
SKO25	Control	Female	85	3:20	Heart failure unspecified (I50.9)
SKO27	Control	Female	76	3:15	Acute vascular disorders of intestine (K55.0)
SKIZ5	Schizophrenia	Female	61	3:59	Heart failure unspecified (I50.9)
SKIZ7	Schizophrenia	Male	57	2:21	Cardiac arrest, cause unspecified (I46.9)
SKIZ8	Schizophrenia	Female	71	3:15	Respiratory arrest (R09.2)
SKIZ9	Schizophrenia	Male	84	2:47	Respiratory arrest (R09.2)
SKIZ10	Schizophrenia	Female	67	3:00	Cardiac arrest, cause unspecified (I46.9)
SKIZ11	Schizophrenia	Female	81	4:20	Heart failure unspecified (I50.9)

### Pre-embedding PV immunofluorescent reactions

The 70-µm-thick sections were cut with a vibratome from blocks obtained from the right DLPFC Area 9 of control (*n* = 8) and schizophrenia (*n* = 6) subjects. Sections were washed in 0.1 M PB and then blocked in 10% normal goat serum (NGS) made up in Tris-buffered saline (TBS; containing 0.2% Triton X-100), pH 7.4, followed by incubation in rabbit anti-parvalbumin (1:2,000, Thermo Fisher Scientific, #PA1-933, RRID: AB_2173898) antibody diluted in TBS containing 2% NGS and 0.2% Triton X-100. Following several washes in TBS, sections were incubated in Cy3-conjugated goat anti-rabbit (1:500, Jackson ImmunoResearch Laboratories). Sections were then mounted on slides in VECTASHIELD (Vector Laboratories). Images covering all layers of DLPFC Area 9 (1 × 2 mm) were taken with a confocal laser scanning microscope (FV3000, Olympus Europe) using a 10× objective. The following image analysis was conducted in Fiji: to create somatic masks, a Gaussian filter (radius, 2) was applied to the images, followed by automatic thresholding (triangle) to detect cell bodies of parvalbumin (PV) immunolabeled cells. These masks were then refined through manual revision. Mean PV intensities of cell bodies were measured in the unfiltered images after background subtraction. For background subtraction, all PV-positive processes were detected using automatic thresholding (mean) of unfiltered images, and a mask was created to cover neuropil areas lacking PV fluorescence. Mean background intensity was measured using this mask and subtracted from the mean somatic PV intensities.

### Tissue preparation for postembedding immunofluorescent reactions

Sample preparation is summarized in Figure 2. Postmortem blocks from the right DLPFC Area 9 of control (*n* = 9) and schizophrenia (*n* = 6) subjects were sectioned into 500-µm-thick slices with a vibratome. Small trapezoid blocks encompassing Layers 1–6 were removed and collected in glass vials. Samples were rinsed in 50% ethanol and dehydrated through a graded series of ethanol (50, 70, 90, 100, 100%), followed by infiltration with acetonitrile. Each dehydration step involved 90 s of microwave irradiation at 850 W, to accelerate dehydration, with vials kept on ice to prevent overheating. Samples were then infiltrated with a 1:1 mixture of acetonitrile and epoxy resin (Durcupan) for 1 h at room temperature before the samples were transferred to pure Durcupan overnight. The small trapezoidal blocks were individually transferred into a flat plastic embedding mold and precisely aligned side-by-side under a stereo microscope. Each Durcupan-infiltrated block was carefully adhered to the preceding one in the correct position. Finally, the mold was slowly filled with Durcupan resin. Samples from control and schizophrenia subjects were embedded in alternating pairs to create sandwich blocks. Fifteen subjects (9 control and 6 schizophrenia) were included in the study, the tissues from which were embedded into two sandwich blocks. The 200–400-nm-thin sections were cut using a Histo Jumbo diamond knife (DiATOME) and mounted onto SuperFrost Ultra Plus glass slides in some experiments in Figures 6 and 7 or onto gelatin-coated coverslips (Figs. 3–9) and left on a hotplate at 60°C for 30 min and then at 80°C overnight. We found no difference in the section adherence properties between the glass slides and coverslips. Signal detection was slightly improved when gelatin-coated coverslips were used. Experiments conducted on both coverslips and glass slides were included into our study. Mounted sections were stored prior to the immunoreactions for up to several weeks at room temperature.

### Postembedding immunofluorescent labeling

Etching of the resin, antigen retrieval, immunolabeling, and elution were carried out as reported previously (Holderith et al., 2020) with slight modifications. The resin was etched with Na-ethanolate for 6 min and rinsed in 96% ethanol three times and then with distilled water. Antigen retrieval was carried out in 0.02 M Tris base, pH 9, containing 0.5% sodium dodecyl sulfate (SDS) for 15 min in a microwave oven at 850 W. After several washes in TBS containing 0.1% Triton X-100 (TBST), pH 7.6, sections were blocked in TBST containing 6% BlottoA (Santa Cruz Biotechnology), 10% NGS (Vector Laboratories), and 1% BSA (Sigma-Aldrich) for 30 min and then incubated in primary antibodies diluted in blocking solution (1:200) at room temperature overnight. All primary antibodies used in the study are listed in Table S1. After several washes, sections were incubated in secondary antibodies diluted (1:200) in TBST containing 10% of the blocking solution for 2 h at room temperature. All secondary antibodies used in the study are listed in Table S1. After labeling, sections were washed and mounted in Slowfade Diamond Antifade mounting medium (Thermo Fisher Scientific). High-magnification images were taken with confocal microscopes using a 60× oil immersion objective (FV3000, 1.35 NA, Olympus Europe or Abberior Instruments Facility Line, 1.42 NA, Abberior Instruments). STED images were taken with an Abberior Instruments Facility Line STED microscope (60×, 1.42 NA oil immersion objective; pixel size, 20 nm). Within the same experiment, all images were taken with the same laser power and detector settings from all control and schizophrenia subjects. Immunoreactions with mouse and rabbit antibodies raised against different epitopes of the GluN1 subunit showed overlapping labeling patterns (Fig. S1).

### Sequential multiplexed postembedding immunofluorescent labeling

For sequential multiplexed labeling (Figs. 4, 5, and 9), each imaging step was followed by an antibody elution step. The immunolabeling was removed through a 5 min incubation in TBST containing 1% SDS, pH 7.7, at 80°C. This elution step has been shown to efficiently abolish >98% of the immunolabeling (Holderith et al., 2020). After a 5 min wash in TBST, a new round of immunolabeling was performed. In each round, an antibody targeting one of the selected synaptic proteins (Munc13-1, GluA2, VGLUT1, and Bassoon) was mixed with antibodies labeling Kv2.1 and PSD-95 to identify the same region and synapses, respectively. Kv2.1, PSD-95, and the selected synaptic proteins were visualized by secondary antibodies conjugated to Alexa Fluor 488, Abberior STAR 580, and Abberior STAR 635P, respectively. Sections were again mounted in Slowfade Diamond on gelatin-coated coverslips. Confocal and STED images were acquired with the Abberior Instruments Facility Line STED microscope. First, confocal images (60×, 1.42 NA objective) were taken of a large field of view (pixel size, 100 nm); then STED images were taken of smaller areas (pixel size, 20 nm). Images collected of the same areas through the four rounds were aligned in Fiji. Images were taken with the same acquisition settings through the four rounds.

### Confocal image analysis of the postembedding immunofluorescent reactions in Layer 2

Confocal images were taken in Layer 2 covering 70 × 210 µm areas in each subject per experiment from 200-nm-thick sections. Images taken in two channels (ch1, PSD-95; ch2, paired synaptic protein) were analyzed in Fiji. First, a binary synaptic mask was created for excitatory synapses based on the fluorescent signal for PSD-95. A Gaussian filter (radius, 2 px) was applied to the PSD-95 channel, followed by an automatic Otsu thresholding to create a mask for the PSD-95 signal. To minimize the joint detection of nearby synapses, we created a second segmentation mask using the Find Maxima function (output, segmented particles). Combination of the two binary masks resulted in the final synaptic mask for measurements on both channels, such as the first channel of PSD-95 and the second channel of the paired synaptic protein (GluA2, AMPA, GluN1, GluN2B, Munc13-1, and Bassoon). The area and intensity measurements were performed in background-subtracted images. The background was determined by the rolling ball algorithm (radius, 7 px). To account for labeling variabilities between reactions, the mean fluorescence of paired synaptic proteins on ch2 were normalized by the population mean of PSD-95 fluorescence and presented as normalized mean intensity. Centroid coordinates of each synapse were used to determine the nearest neighbor distances (NNDs). To minimize the influence of cell bodies, fractures, and the variable distribution of blood vessels on our measurement of synaptic density, we determined the mean NNDs between synaptic puncta to characterize the abundance of synapses within the samples. Synapse density was also determined by calculating the number of synapses per measured area.

### STED analysis of the Munc13-1 nanoclusters

PSD-95 and Munc13-1 were colabeled in a postembedding immunofluorescent reaction carried out on 300-nm-thin resin embedded sections of nine control and six schizophrenia subjects. This increased section thickness (compared to 200 nm) was chosen to improve the likelihood of finding en face synapses. PSD-95 and Munc13-1 were visualized by Abberior STAR 580- and Abberior STAR 635P-coupled secondary antibodies, respectively. STED images were taken at high magnification (60×, 1.42 NA objective; pixel size, 20 nm) from Layer 2 of the right DLPFC Area 9. Synaptic masks were created based on the PSD-95 fluorescence as described above. For the detection of Munc13-1 nanoclusters, synaptic masks were dilated by 3 px to include presynaptic AZs of side view synapses. Synaptic areas were measured using nondilated synaptic masks. Synapses with elongated PSD-95 fluorescence and a slightly laterally shifted Munc13-1 fluorescent signal were classified as “side view” synapses. Synaptic masks with spatially expanded PSD-95 fluorescence, exhibiting a round, oval, or irregular shape with overlapping Munc13-1 fluorescence, were classified as “en face” synapses. En face synapses either displayed perforated or nonperforated shapes. Synapses were also characterized by their shape index, the aspect ratio (AR), calculated as the ratio of the major axis length to the minor axis length of a fitted ellipse. We used an empirically determined AR threshold for separating side view (AR ≥ 2.7) and en face (AR < 2.7) synapses. PSD-95 fluorescence distributed homogeneously within the synapses, while the Munc13-1 signal formed distinct, high-intensity puncta, consistent with the formation of Munc13-1 nanoclusters in the presynaptic AZ described in mice (Sakamoto et al., 2018; Rebola et al., 2019; Karlocai et al., 2021; Aldahabi et al., 2022). Munc13-1 intensity peaks were detected and counted in Fiji using the Find Maxima function (output: point selection). The same threshold was used for all subjects.

### Sequential labeling and confocal analysis of AMPAR, GluN1, and GluN2B content of excitatory synapses targeting PV immunopositive (PV+) dendrites in deeper layers

We performed two rounds of triple-labeling reactions on 400-nm-thin resin–embedded sections mounted on gelatin-coated coverslips in two sets of experiments, as detailed in Table S2. The 400 nm section thickness ensured that larger fractions of individual PV dendrites were included within single sections. In the first set of experiments, we labeled PV, AMPARs (using the pan-AMPA antibody), and GluN1 in the first round with a mixture of rabbit polyclonal, guinea pig polyclonal, and mouse monoclonal antibodies (1:200), respectively, and visualized the reactions with Alexa Fluor 488-, Cy3-, and Alexa Fluor 647-coupled secondary antibodies, respectively. Low-magnification confocal images (20× objective, Olympus FV3000) were taken of each section to create a map. PV+ processes were identified, and high-magnification three–channel images were taken (60×, 1.35 NA objective, Olympus FV3000). Following antibody elution, the sections were relabeled in a second round. A mixture of rabbit polyclonal anti-PV, guinea pig polyclonal anti-PSD-95, and a mouse monoclonal anti-myelin basic protein (MBP) antibody was applied, and subsequently visualized by Alexa 488-, Cy3-, and Alexa 647-coupled secondary antibodies, respectively. MBP labeling was used to exclude thick, myelinated PV+ axons. The lack of synaptic coverage served as an additional criterion to distinguish these axons from PV+ dendrites. In the second experiment, we labeled MBP, PV, PSD-95, and GluN1 in the first round and MBP, GluN2B, and AMPARs in the second round as described in Table S2. All subjects were screened, but we failed to collect a sufficient number of PV+ dendrites in two control (SKO7 and SKO13) and in one schizophrenia (SKIZ9 in Exp1 and SKIZ7 in Exp2) subjects. Images were aligned by the PV signal in the first experiment and by the MBP signal in the second experiment. Images were aligned with the Linear Stack Alignment with SIFT Multichannel plugin and analyzed in Fiji. PSD-95 fluorescence was less intense in synapses on PV+ dendrites compared with the surrounding synapse population in line with findings in rodent (Micheva et al., 2025). To facilitate the reliable detection of these synapses, synaptic masks were created based on the summed fluorescence of PSD-95, AMPA, and GluN1. PV+ dendrites were identified and manually outlined to create a PV mask (PV dendritic mask). Synaptic masks overlapping the PV dendritic mask were considered as synapses on PV+ dendrites. All synapses containing PSD-95 signal (>10 integrated PSD-95 fluorescence) were analyzed. Density of excitatory synapses on PV+ dendrites was measured in 10.7 ± 9 and 8.8 ± 6.7 dendrites per subject in control and schizophrenia subjects, respectively. Mean PSD-95 content represents the average of integrated PSD-95 fluorescence measured in individual synapses in each subject. Synaptic AMPAR, GluN1, and GluN2B densities are calculated as the ratio of fluorescent integrals of AMPAR (or GluN1 or GluN2B) and PSD-95 in each synapse. Synaptic AMPAR, GluN1, or GluN2B densities on PV+ dendrites are given relative to the population values of the surrounding synapses. Data for PSD95, AMPAR, and GluN1 analysis were acquired from two experiments (CON, *n* = 7; SCZ, *n* = 6), while data for GluN2B analysis were obtained from a single experiment (CON, *n* = 7; SCZ, *n* = 5).

### Experimental design and statistical analysis

**Figure 1.**
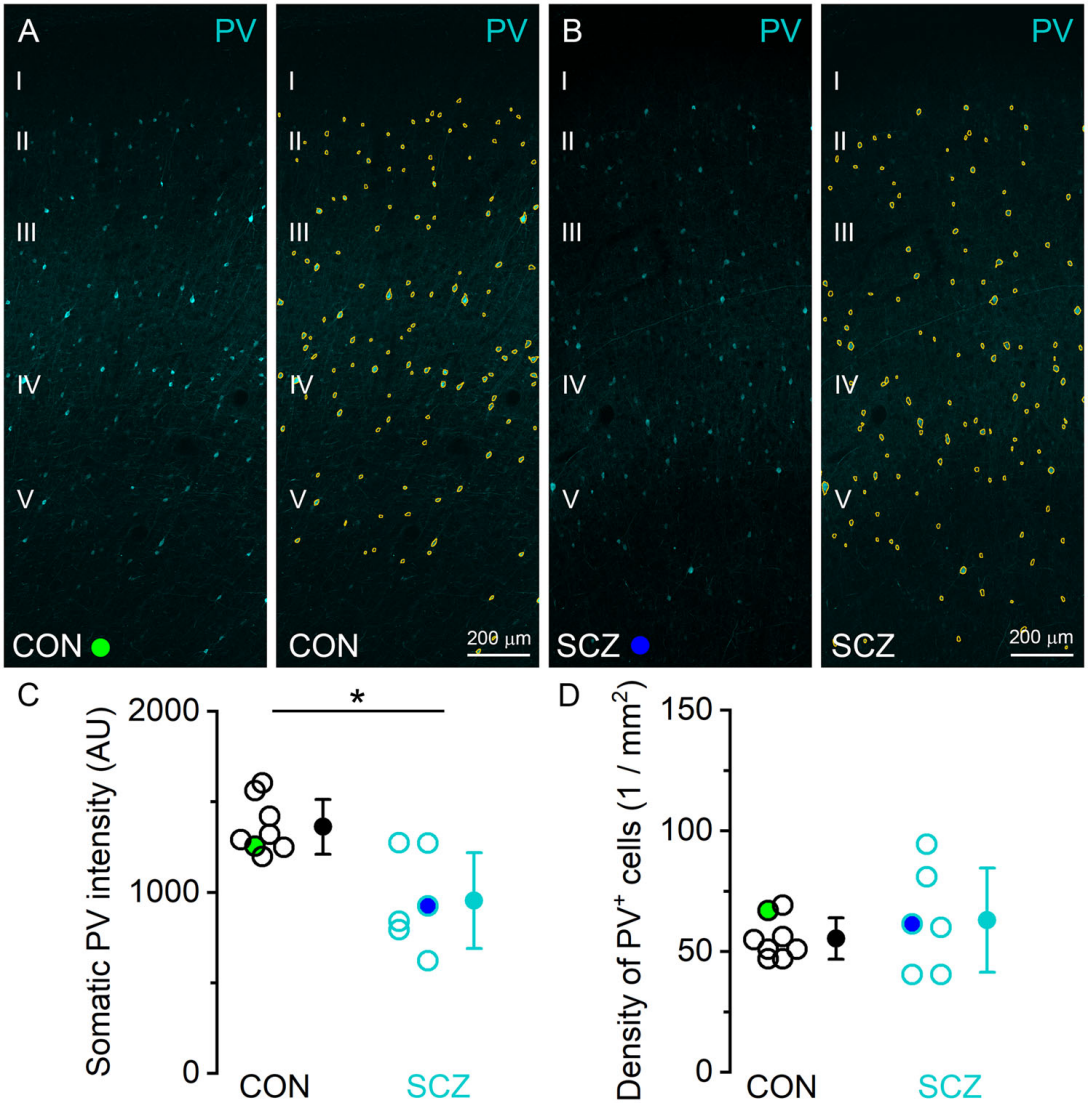
Reduced PV immunoreactivity in PV-expressing INs in DLPFC Area 9 of schizophrenia subjects. ***A***, ***B***, Pre-embedding immunofluorescent reaction for PV in DLPFC Area 9 in a control (***A***, CON) and a schizophrenia (***B***, SCZ) subject. PV immunopositive cell bodies included in the analysis are outlined in orange (***A*** right and ***B*** right). ***C***, Mean somatic PV intensity is reduced by 30% (*p* = 0.007, unpaired *t* test with HBC) in SCZ (955 ± 265; *n* = 6) subjects compared with CON (1,362 ± 151; *n* = 8) subjects. ***D***, The density of PV immunopositive cells does not differ (*p* = 0.380, unpaired *t* test with HBC) between CON (55.4 ± 8.5 μm; *n* = 8) and SCZ (63 ±21.6 μm; *n* = 6) subjects. Open circles represent mean data from individual subjects; filled circles indicate population mean ± SD.

Perfusion-fixed blocks from the DLPFC of nine control and six schizophrenia subjects were used in this study. The Kolmogorov–Smirnov and Shapiro–Wilk tests were used for testing our data for normality on large (Figs. 6*G*, 8*C*) and small datasets (Figs. 1*C*–*D*, 6*B*–*F*, 7, 8*B*, 9*B*–*E*), respectively. Correlations were determined with Spearman's rank correlation (Figs. 6*G*, 8*C*); *p* values were calculated from two-tailed *t* test. For multiple-comparison unpaired *t* test was used with post hoc Holm–Bonferroni’s correction (HBC). General linear regression analysis was used to analyze the effects of age, gender, and PMI. Statistical analyses were performed in OriginPro (2020B). Statistical significance was assessed at *p* < 0.05. Data are presented as mean ± SD throughout the manuscript. “*n*” represents either the number of subjects or the number of synapses per subject (mean ± SD), unless stated otherwise.

### Data accessibility

The data used in this study are available from the corresponding author upon request.

### Code accessibility

The custom-written scripts used to analyze the data in this study are available from the corresponding author upon request.

## Results

### Reduced PV immunoreactivity in PV-expressing interneurons in DLPFC Area 9 of schizophrenia subjects

To characterize our samples from schizophrenia subjects, we first aimed to investigate previously identified molecular alterations associated with schizophrenia. Previous studies investigating the DLPFC Area 9 of schizophrenia subjects have reported reduced expression of PV mRNA (Hashimoto et al., 2003; Fung et al., 2010; Volk et al., 2012) and reduced PV immunoreactivity (Belforte et al., 2010; Glausier et al., 2014; Enwright et al., 2016) in the soma of PV-expressing GABAergic interneurons (INs), without any change in their density (Hashimoto et al., 2003; Chung et al., 2016; Batiuk et al., 2022). We performed immunofluorescent reactions for PV in sections obtained from the right DLPFC Area 9 of postmortem, perfusion-fixed control and schizophrenia subjects. Besides neuropil labeling, PV fluorescence was the most intense in somata and dendrites of INs in Layer 3 and 4, but PV immunopositive (PV+) INs were also detected in superficial and deeper layers in both control and schizophrenia subjects (Fig. 1*A*,*B*). To exclude mixed fluorescent signals in the neuropil originating from GABAergic local circuit and thalamic axons, we outlined PV+ cell bodies and measured somatic PV immunofluorescent intensities (Fig. 1*C*). In our reactions, the mean somatic PV intensity was ∼30% lower in schizophrenia (*p* = 0.007, unpaired *t* test with HBC), without any detectable alteration in their densities (Fig. 1*D*; *p* = 0.380, unpaired *t* test with HBC). Importantly, general linear regression analysis revealed that somatic PV intensities were not affected by age (*p* = 0.190), PMI (*p* = 0.193), or gender (*p* = 0.204). Having confirmed that a well known molecular alteration also characterizes our samples, we processed the tissue further for high-resolution molecular characterization of excitatory synapses.

### Molecular characterization of excitatory synapses using multiplexed postembedding immunofluorescent reactions

**Figure 2.**
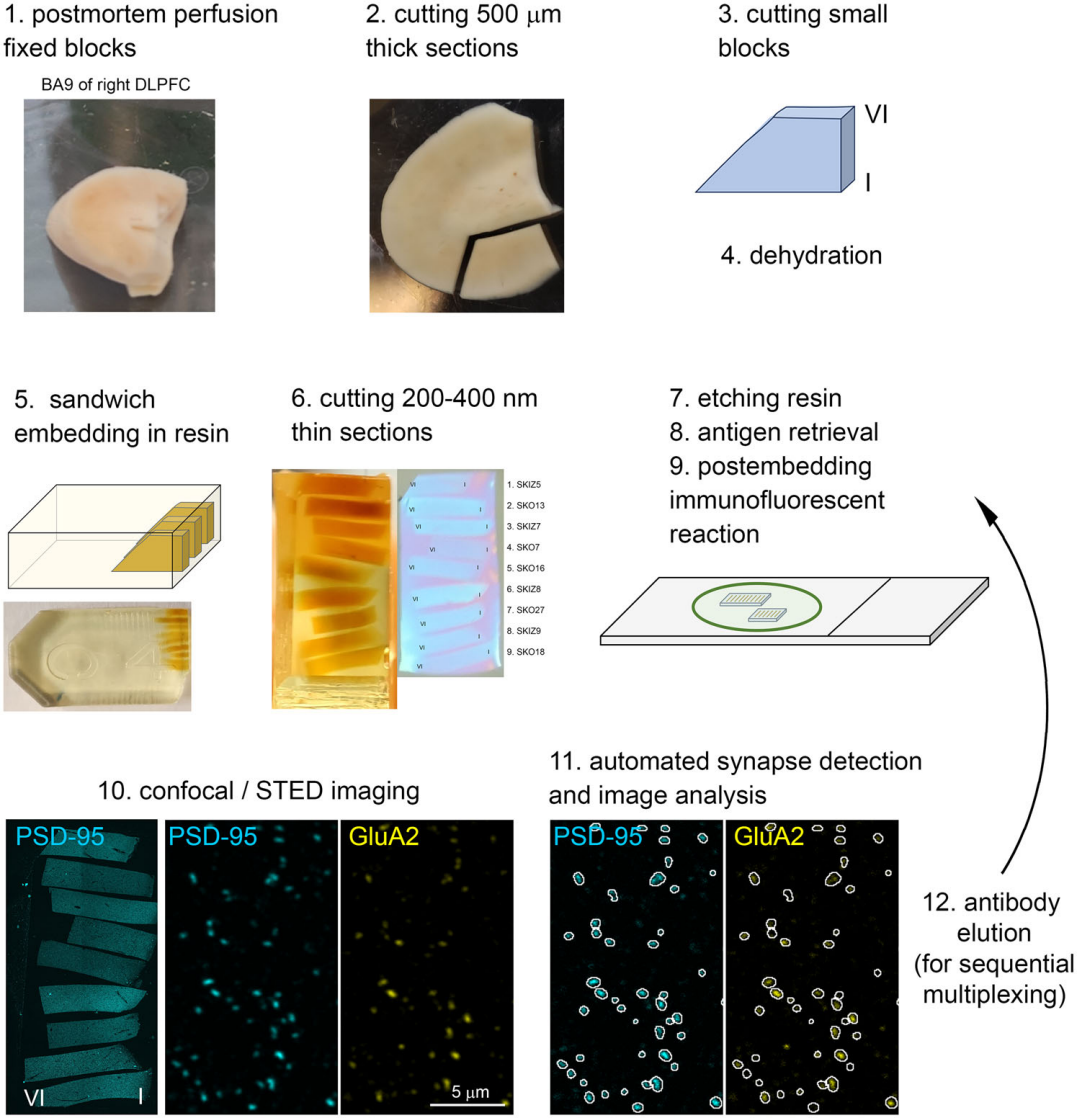
Sample preparation and labeling workflow for multiplexed postembedding immunofluorescent reactions. Postmortem fixed blocks (1) from the right DLPFC Area 9 were sectioned (2) into 500-μm-thick slices with a vibratome. Small trapezoid blocks encompassing all cortical layers (3) were dehydrated (4). Samples from control and schizophrenia subjects were embedded in alternating pairs in epoxy resin (5). Fifteen subjects were included in the study, embedded in two sandwich blocks. Ultrathin sections (200–400 nm) were cut and mounted on frosted glass slides or gelatin-coated coverslips (6). To enhance the labeling efficiency, resin etching (7) and microwave irradiation-based antigen retrieval steps (8) were performed prior to postembedding immunofluorescent labeling (9) for synaptic proteins. Synaptic labeling was visualized using confocal or STED microscopy (10), and images were acquired from each subject. Automated synapse detection and image analysis (11) were conducted in Fiji (see Materials and Methods). For sequential multiplexing, imaging was followed by an antibody elution step (12) before the next labeling round.

We employed a modified version of a multiplexed postembedding immunofluorescence method (Fig. 2) recently developed in our laboratory (Holderith et al., 2020) and successfully used in rodent brains for molecular characterization of excitatory synapses (Karlocai et al., 2021). Tissue blocks were obtained from postmortem perfusion-fixed brains (Table 1) with short PMI (3.3 ± 0.7 h) and uniform fixation conditions. Five-hundred-micrometer-thick sections were cut, dehydrated, and embedded in epoxy resin (Durcupan) before being resectioned at 200–400 nm thickness (Fig. 2). We have modified our standard protocol with a step in which the ultrathin sections were subjected to microwave irradiation for 15 min in 0.5% SDS solution, which dramatically increased the immunofluorescent labeling of most examined proteins.

**Figure 3.**
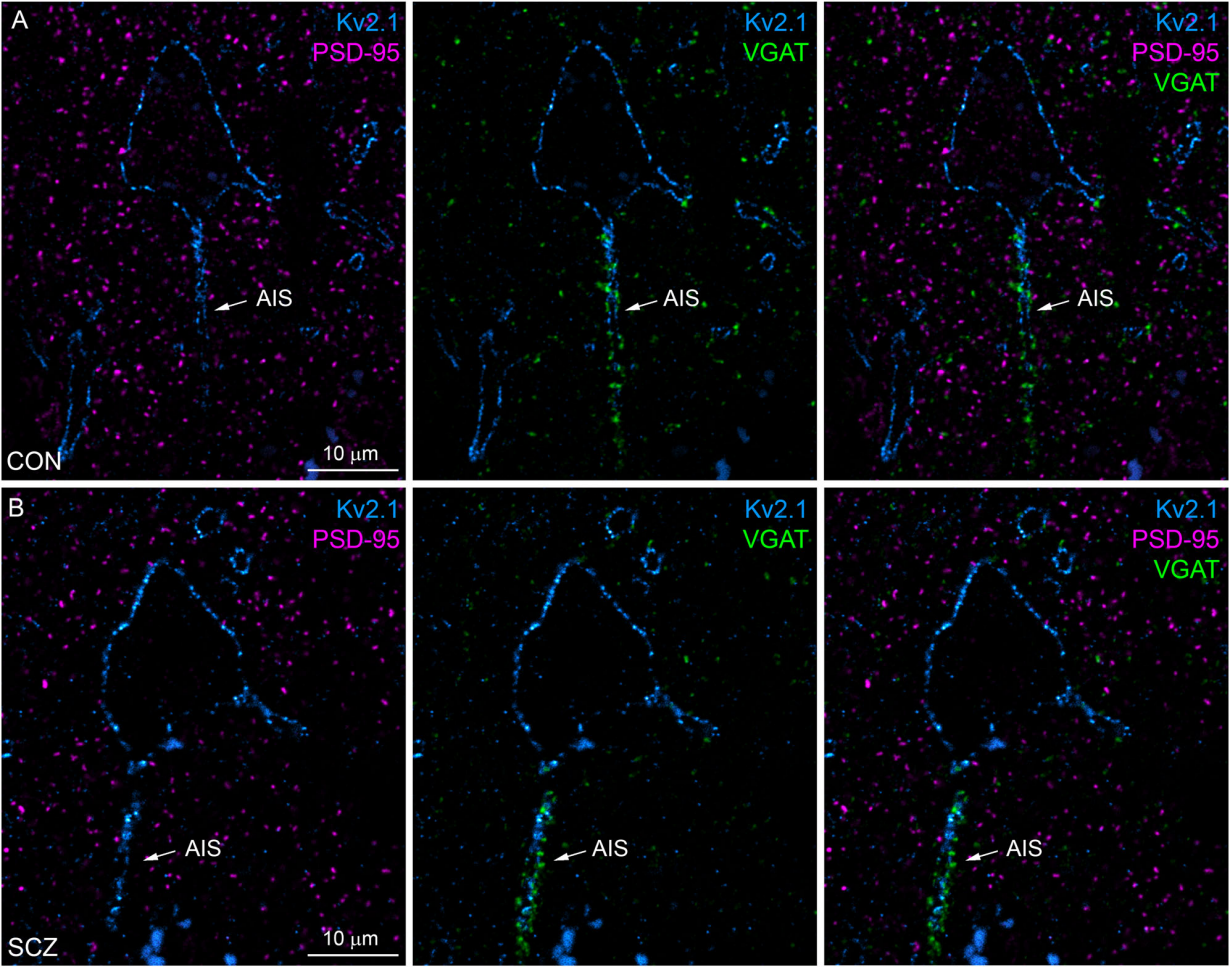
Immunofluorescent localization of VGAT and PSD-95 in the human prefrontal cortex. ***A***, ***B***, Confocal images showing triple immunofluorescent labeling of a 400-nm-thin resin–embedded section for Kv2.1 (light blue), PSD-95 (magenta), and VGAT (green) in a human control (***A***, CON) and a schizophrenia (***B***, SCZ) subject in Layer 2 of DLPFC Area 9. VGAT immunopositive puncta are sparsely distributed in the neuropil, but they densely cover the axon initial segments (AIS, arrow) of Kv2.1 labeled PCs, without any overlap with PSD-95-immunopositive puncta.

**Figure 4.**
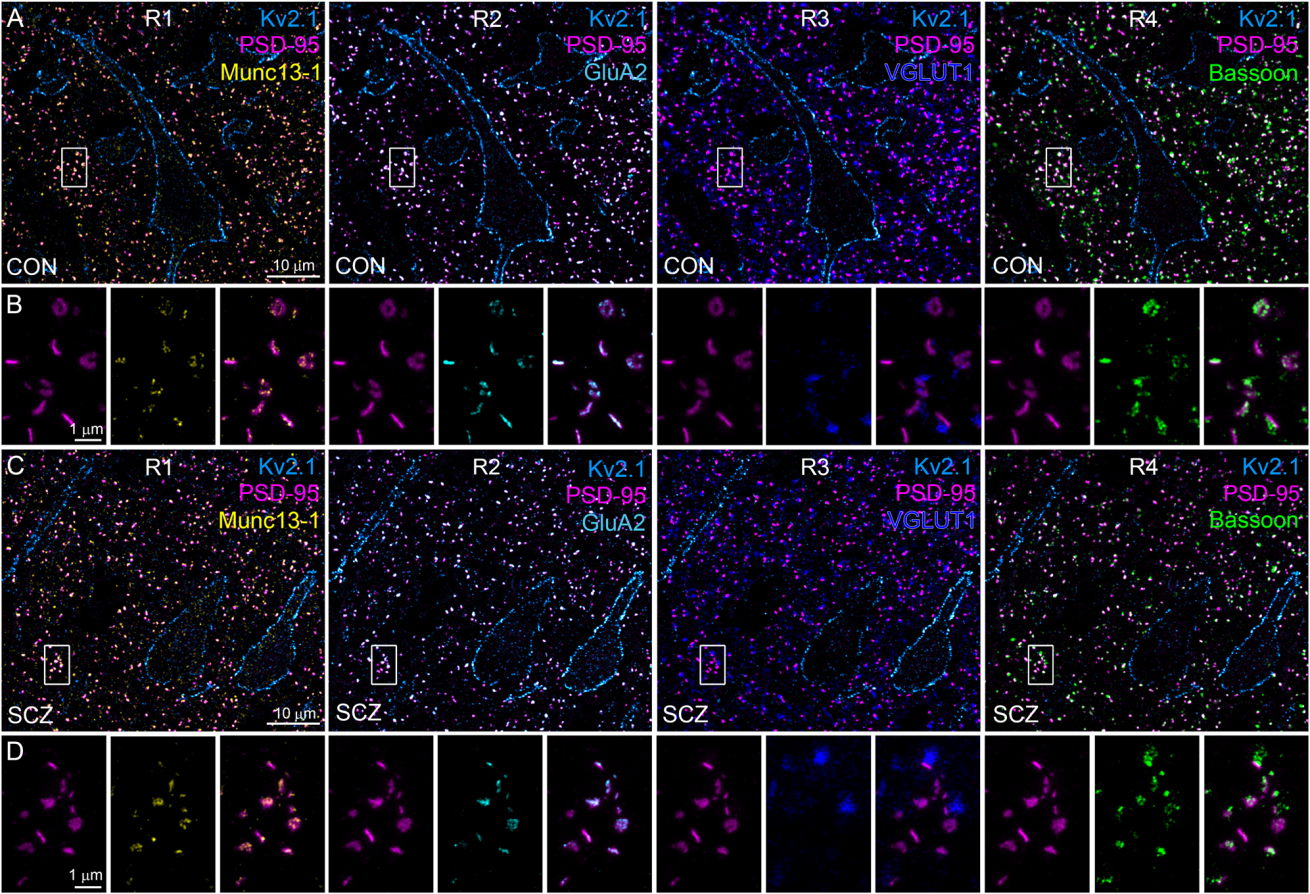
Molecular characterization of excitatory synapses in the human prefrontal cortex using multiplexed immunolabeling. ***A***, Confocal images showing multiple immunofluorescent labeling of a 300-nm-thin resin–embedded section obtained from Layer 2 of DLPFC Area 9 of a control (CON) subject. Four rounds (R1-R4) of triple labeling were performed to detect Munc13-1 (yellow, R1), GluA2 (cyan, R2), VGLUT1 (blue, R3), and Bassoon (green, R4; always visualized with Abberior STAR 635P) in the same set of excitatory synapses. In each round, Kv2.1 (light blue, Alexa Fluor 488) and PSD-95 (magenta, Abberior STAR 580) were also immunolabeled. ***B***, High-magnification STED images of the same synapses shown in the insets (white boxes) in panel ***A***. ***C***, ***D***, Same as ***A*** and ***B***, but images were obtained from a schizophrenia (SCZ) subject.

**Figure 5.**
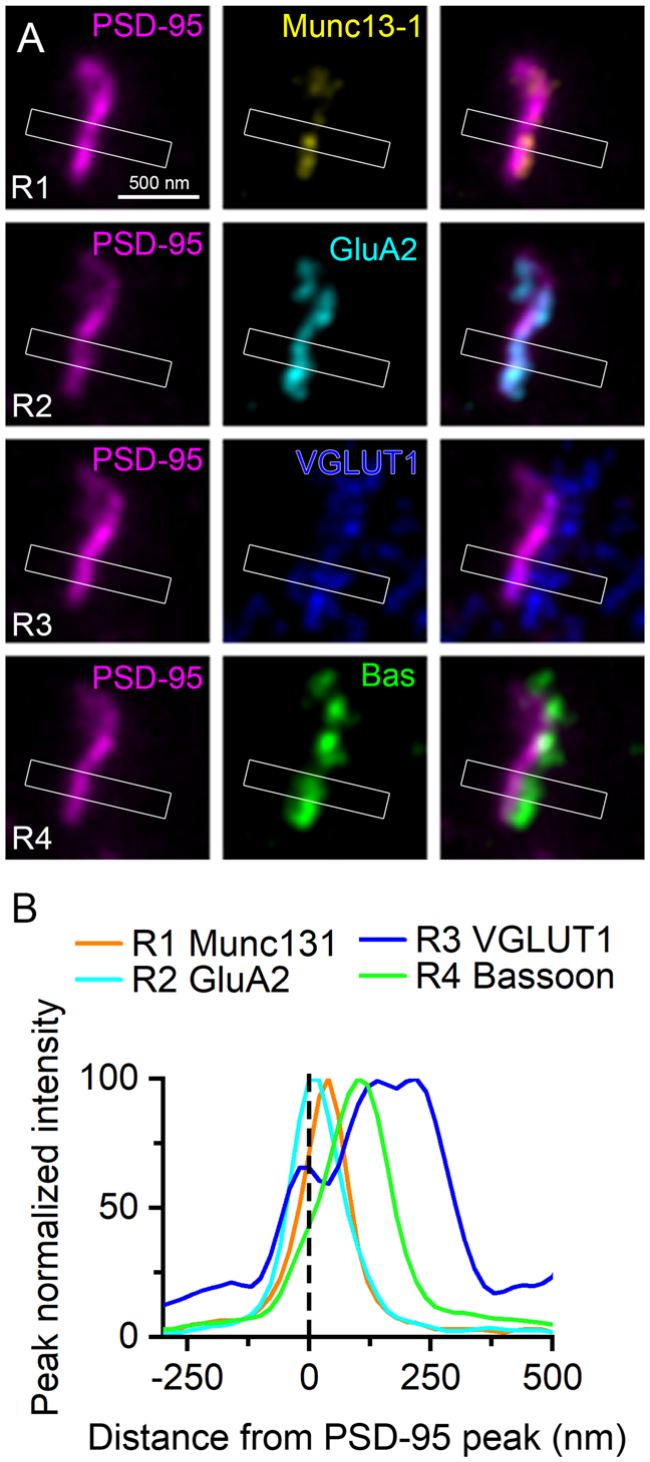
Lateral spatial distribution of pre- and postsynaptic proteins in a PSD-95 immunopositive human excitatory synapse. ***A***, STED images showing the side view of an excitatory synapse in Layer 2 of the human DLPFC Area 9 of a control subject. The synapse was sequentially labeled for Munc13-1 (yellow), GluA2 (cyan), VGLUT1 (blue), and Bassoon (green) in four rounds (R1-R4). Kv2.1 (data not shown) and PSD-95 (magenta) were also labeled to identify the same region and synapses in each round. ***B***, Peak normalized intensity plots from the boxed areas (860 × 175 nm) shown in ***A***. The distributions of immunoreactivity for synaptic proteins are shown relative to the peak of PSD-95 signal (dashed line). Peak distances, 0 nm (GluA2), 40 nm (Munc13-1), 100 nm (Bassoon), and 220 nm (VGLUT1).

First, we looked for a molecular marker that labels potentially every excitatory synapse of the human cortex. PSD protein PSD-95 is selectively expressed in vast majority of cortical glutamatergic synapses in mice, where its number shows a tight correlation with the synaptic size (Karlocai et al., 2021; Micheva et al., 2025). Punctate immunoreactivity for PSD-95 was present in all layers of the DLPFC and, as expected, did not overlap with labeling for vesicular GABA transporter (VGAT; Fig. 3). To characterize the molecular composition of PSD-95–labeled excitatory synapses, we used multiplexed postembedding immunofluorescent labeling for PSD-95, presynaptic AZ proteins Munc13-1 and Bassoon, presynaptic vesicular glutamate transporter 1 (VLGUT1), and the postsynaptic AMPAR subunit GluA2 (Fig. 4). Following each labeling round, we imaged the reactions with confocal and STED microscopy and then performed an elution step (see Materials and Methods) before restaining and reimaging. Kv2.1 and PSD-95 labeling were used to identify the same region and set of synapses, respectively, in every round. Kv2.1 labeling strongly outlined the plasma membranes of pyramidal cell (PC) bodies and their proximal dendrites in both control and schizophrenia subjects. Punctate PSD-95 fluorescence was distributed in the neuropil surrounding the Kv2.1-labeled PCs, avoiding neuronal cytoplasm and nuclei. Confocal images revealed intense punctate labeling for the tested synaptic proteins, demonstrating colocalizations (Munc13-1, GluA2) and close associations (VGLUT1, Bassoon) with PSD-95 (Fig. 4*A*,*C*) in both control and schizophrenia subjects. High-resolution STED images revealed a finer, more detailed spatial arrangements of these proteins (Figs. 4*B*,*D*, 5). In our ultrathin sections, synapses appear at distinct orientations. In en face or top view orientations, pre- and postsynaptic membranes are on top of one another resulting in apparent colocalization of pre- and postsynaptic proteins. In contrast, when synapse orientation is perpendicular to the cutting plane (side view), pre- and postsynaptic labeling appears as thin parallel lines separated by a small gap that corresponds to the synaptic cleft. In our reactions, side view synapses were characterized by an elongated PSD-95 fluorescence, while en face synapses exhibited spatially expanded PSD-95 fluorescence with a round, oval, or irregular shape (e.g., C-shape or perforated) in both control and schizophrenia subjects (Fig. 4*B*,*D*). Peak-aligned intensity distributions (Fig. 5) demonstrated increasing lateral distances of the various synaptic proteins from the postsynaptic PSD-95, with GluA2 being the closest, followed by the Munc13-1 and Bassoon in the presynaptic AZ, and finally VGLUT1 associated with a large pool of synaptic vesicles inside the presynaptic terminal (Fig. 5*B*). This precise lateral arrangement of pre- and postsynaptic molecules in human excitatory synapses is consistent with that observed in rodents.

Next, we looked at the intrasynaptic distributions of synaptic proteins using STED imaging. Recently, it has been shown that presynaptic vesicle release sites align with postsynaptic receptor clusters in so-called transsynaptic nanocolumns in mouse excitatory synapses (Tang et al., 2016). We observed a uniformly distributed PSD-95 fluorescent signal within synapses, consistent with our previous electron microscopic findings in mouse hippocampal synapses (Karlocai et al., 2021). The homogeneous intrasynaptic PSD-95 distribution, together with the shape and size of the labeled synapses, remained unchanged throughout the four labeling rounds (Fig. 4*B*,*D*), suggesting that the elution steps did not disrupt the synaptic structure and molecular architecture. In mice, Munc13-1 has been suggested to form intrasynaptic clusters within the AZ, representing vesicular release sites (Reddy-Alla et al., 2017; Sakamoto et al., 2018; Rebola et al., 2019; Karlocai et al., 2021; Aldahabi et al., 2022). Similarly, AMPARs can be organized in clusters in some excitatory synapses in mice, but not in others (Masugi-Tokita et al., 2007; Tang et al., 2016; Szoboszlay et al., 2017). Consistent with the findings in rodents, we observed uneven, clustered subsynaptic distribution patterns of Munc13-1 and GluA2 immunofluorescence in both control and schizophrenia subjects. Thus, our method enabled us to visualize the expression and spatial distribution of key synaptic proteins in human excitatory synapses with unprecedented sensitivity and resolution. Next, we aimed to quantify and compare the size, density, and synaptic protein content of excitatory synapses in the prefrontal cortex of control and schizophrenia subjects. We analyzed nearly 800,000 synapses (Table 2) in total throughout the study.

**Table 2.** Summary of data and statistics presented in Figures 6–9

	Control					Schizophrenia					Statistics		Figure
	Population mean	Subject	Reaction	Number of synapses in a subject per reaction	Total number of synapses per subject	Population mean	Subject	Reaction	Number of synapses in a subject per reaction	Total number of synapses per subject			
Measured parameter	Mean ± SD	*n*	*n*	Mean ± SD	Mean ± SD	Mean ± SD	*n*	*n*	Mean ± SD	Mean ± SD	*p*	Test	
Layer 2													
Synaptic density (1/μm^2^)	0.15 ± 0.02	9	21	2,123.6 ± 223	44,596 ± 4,682	0.18 ± 0.05	6	21	2,518 ± 721	52,881 ± 15,136	0.150	Unpaired *t* test	6*B*
Nearest neighbor distance (μm)	1.13 ± 0.05	9	21	2,123.6 ± 223	44,596 ± 4,682	1.09 ± 0.09	6	21	2,518 ± 721	52,881 ± 15,136	0.299	Unpaired *t* test	
Synaptic area (μm^2^)	0.256 ± 0.004	9	21	2,124 ± 223	44,596 ± 4,682	0.255 ± 0.005	6	21	2,518 ± 721	52,881 ± 15,136	0.656	Unpaired *t* test	6*C*
PSD-95 mean intensity (AU)	497 ± 116	9	21	2,124 ± 223	44,596 ± 4,682	470 ± 140	6	21	2,518 ± 721	52,881 ± 15,136	0.689	Unpaired *t* test	6*C*
Synaptic area (μm^2^) of all synapses (STED)	0.11 ± 0.01	9	1	264 ± 93	264 ± 93	0.10 ± 0.02	6	1	294 ± 189	294 ± 189	0.198	Unpaired *t* test	6*E*
PSD-95 mean intensity (AU) in all synapses (STED)	29.9 ± 6.3	9	1	264 ± 93	264 ± 93	32.5 ± 10.1	6	1	294 ± 189	294 ± 189	0.533	Unpaired *t* test	6*E*
Area (μm^2^) of en face synapses (STED)	0.12 ± 0.01	9	1	196 ± 71	196 ± 71	0.10 ± 0.02	6	1	215 ± 149	215 ± 149	0.184	Unpaired *t* test	6*F*
PSD-95 mean intensity (AU) in en face synapses (STED)	29.0 ± 5.7	9	1	196 ± 71	196 ± 71	31.4 ± 9.5	6	1	215 ± 149	215 ± 149	0.557	Unpaired *t* test	6*F*
Normalized GluA2 mean intensity	1.08 ± 0.25	9	3	2,096 ± 245	6,288 ± 735	0.91 ± 0.34	6	3	2,541 ± 830	7,622 ± 2,490	0.571	Unpaired *t* test	7*A*
Normalized AMPAR mean intensity	0.34 ± 0.08	9	5	2,012 ± 293	10,062 ± 1,467	0.38 ± 0.05	6	5	2,384 ± 789	11,920 ± 3,943	0.821	Unpaired *t* test	7*B*
Normalized GluN1 mean intensity	0.36 ± 0.10	9	2	2,816 ± 411	5,632 ± 823	0.39 ± 0.09	6	2	3,203 ± 664	6,405 ± 1,327	0.669	Unpaired *t* test	7*C*
Normalized GluN2B mean intensity	0.46 ± 0.05	9	4	2,199 ± 258	8,794 ± 1,033	0.50 ± 0.06	6	4	2,662 ± 820	10,648 ± 3,278	0.621	Unpaired *t* test	7*D*
Normalized Munc13-1 mean intensity	0.82 ± 0.11	9	4	1,877 ± 213	7,509 ± 854	0.97 ± 0.12	6	4	2,237 ± 695	8,949 ± 2,778	0.187	Unpaired *t* test	7*E*
Normalized Bassoon mean intensity	0.70 ± 0.14	9	3	2,103 ± 182	6,310 ± 547	0.94 ± 0.31	6	3	2,446 ± 694	7,338 ± 2,081	0.285	Unpaired *t* test	7*F*
Number of Munc13-1 nanoclusters in all synapses	3.4 ± 0.5	9	1	264 ± 93	264 ± 93	3.3 ± 0.5	6	1	294 ± 189	294 ± 189	0.861	Unpaired *t* test	8*B*
Number of Munc13-1 nanoclusters in en face synapses	3.4 ± 0.6	9	1	196 ± 71	196 ± 71	3.4 ± 0.5	6	1	215 ± 149	215 ± 149	0.831	Unpaired *t* test	8*B*
Number of Munc13-1 nanoclusters in side view synapses	3.2 ± 0.3	9	1	68 ± 23	68 ± 23	3.2 ± 0.5	6	1	79 ± 45	79 ± 45	0.951	Unpaired *t* test	8*B*
Layers 3 and 4													
Synaptic density (1/μm^2^)	0.23 ± 0.02	7	2	4,489 ± 3,069	8,977 ± 6,139	0.23 ± 0.04	6	2	3,714 ± 2,410	6,820 ± 5,441	0.723	Unpaired *t* test	
Synaptic PSD-95 integral (AU) (PV)	122 ± 22	7	2	169 ± 127	337 ± 255	126 ± 38	6	2	128 ± 112	243 ± 237	0.829	Unpaired *t* test	9*B*
Synaptic PSD-95 integral (AU) (non-PV)	192 ± 41	7	2	4,321 ± 2,944	8,641 ± 5,889	190 ± 82	6	2	3,587 ± 2,307	6,578 ± 5,221	0.951	Unpaired *t* test	9*B*
Normalized synaptic AMPAR density (PV)	1.72 ± 0.14	7	2	169 ± 127	337 ± 255	1.54 ± 0.25	6	2	128 ± 112	243 ± 237	0.116	Unpaired *t* test	9*C*
Normalized synaptic GluN1 density (PV)	1.60 ± 0.15	7	2	169 ± 127	337 ± 255	1.30 ± 0.19	6	2	128 ± 112	243 ± 237	0.031	Unpaired *t* test	9*D*
Normalized synaptic GluN2B density (PV)	1.72 ± 0.25	7	1	185 ± 108	185 ± 108	1.43 ± 0.21	5	1	149 ± 114	149 ± 114	0.117	Unpaired *t* test	9*E*
Density of synapses along PV dendrites (1/μm)	0.87 ± 0.17	7	1	153 ± 162	153 ± 162	0.94 ± 0.22	5	1	142 ± 119	142 ± 119	0.524	Unpaired *t* test	

### Preserved synaptic density, size, and PSD-95 content in Layer 2 of DLPFC Area 9 of schizophrenia subjects

**Figure 6.**
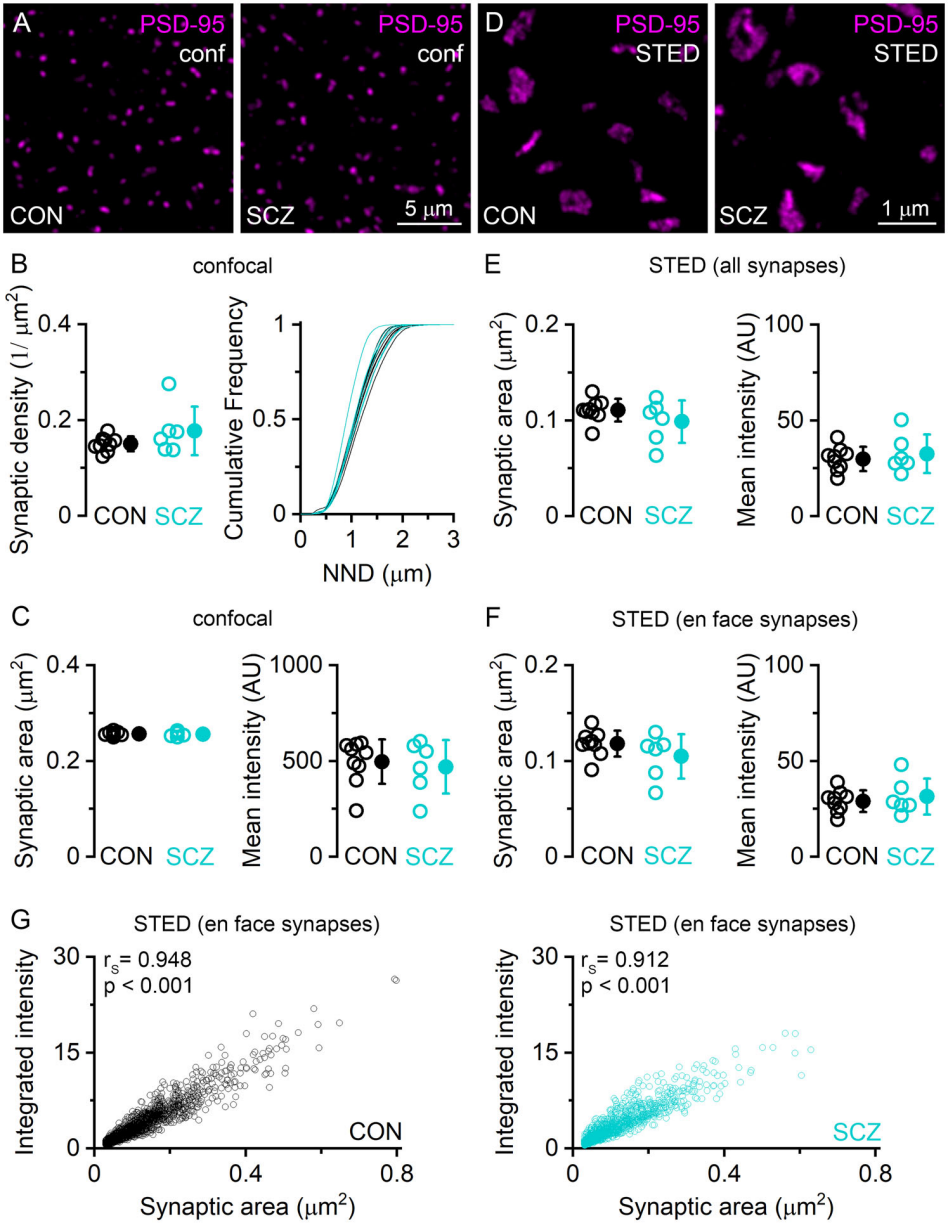
Similar density, size, and PSD-95 content of excitatory synapses in DLPFC Area 9 in control and schizophrenia subjects. ***A***, Confocal images (conf) of Layer 2 of DLPFC Area 9 of control (CON) and schizophrenia (SCZ) subjects immunolabeled for PSD-95. ***B***, Mean density of PSD-95 puncta is similar (*p* = 0.15, unpaired *t* test) in CON and SCZ subjects (left). Cumulative probability plots show overlapping distributions of NNDs in CON and SCZ subjects (right). ***C***, Quantitative analysis in excitatory synapses does not reveal differences in synaptic size (*p* = 0.656, unpaired *t* test) or mean PSD-95 intensity (*p* = 0.689, unpaired *t* test) between CON (*n* = 9) and SCZ (*n* = 6) subjects. ***D***, STED images of excitatory synapses labeled for PSD-95 in Layer 2 of DLPFC Area 9. ***E, F***, Quantitative analysis of all (***E***) and en face (***F***) excitatory synapses does not reveal any difference in size (***E***, *p* = 0.198; ***F***, *p* = 0.184, unpaired *t* test) or mean PSD-95 intensity (***E***, *p* = 0.533; ***F***, *p* = 0.557, unpaired *t* test) between CON (*n* = 9) and SCZ (*n* = 6) subjects. ***G***, Correlations between integrated PSD-95 intensity and synaptic area within en face synapses in CON (left) and SCZ (right) subjects. Data were pooled from all individuals (CON, *n* = 1,762 synapses from 9 subjects; SCZ, *n* = 1,291 synapses from 6 subjects). *r*_S_, Spearman's rank correlation coefficient. Open circles in ***B, C, E,*** and ***F*** represent mean data from individual subjects; filled circles indicate population mean ± SD.

We investigated the abundance and size of excitatory synapses by making quantitative evaluation of PSD-95–labeled synaptic puncta in Layer 2 of DLPFC Area 9. We determined synaptic densities (Table 2) and the mean NNDs between synaptic puncta (Fig. 6*B*), which is also a reliable measure of synapse density (Szoboszlay et al., 2017). The mean synapse density was not significantly different between control and schizophrenia subjects (*p* = 0.15, unpaired *t* test). Distributions of NNDs of synapses in each individual subject also overlapped between control and schizophrenia samples. The synaptic area and mean PSD-95 intensity were similar between the two conditions in our confocal reactions (Fig. 6*C*). Next, we analyzed synapses on images obtained with STED microscopy (Fig. 6*D*–*G*). There were no significant differences in the synaptic area and mean PSD-95 intensity between control and schizophrenia subjects regardless whether all synapses (area, *p* = 0.198; mean intensity, *p* = 0.533; unpaired *t* test) or only en face synapses (area, *p* = 0.184; mean intensity, *p* = 0.557; unpaired *t* test) were analyzed. The integrated intensity of PSD-95 fluorescence showed equally strong correlation with the synaptic area in both control (*r*_S_, 0.948; *p* < 0.001) and schizophrenia (*r*_S_, 0.912; *p* < 0.001) subjects (Fig. 6*G*). All data are provided in Table 2.

### Excitatory synapses in DLPFC Area 9 contain comparable levels of key synaptic molecules in control and schizophrenia subjects

**Figure 7.**
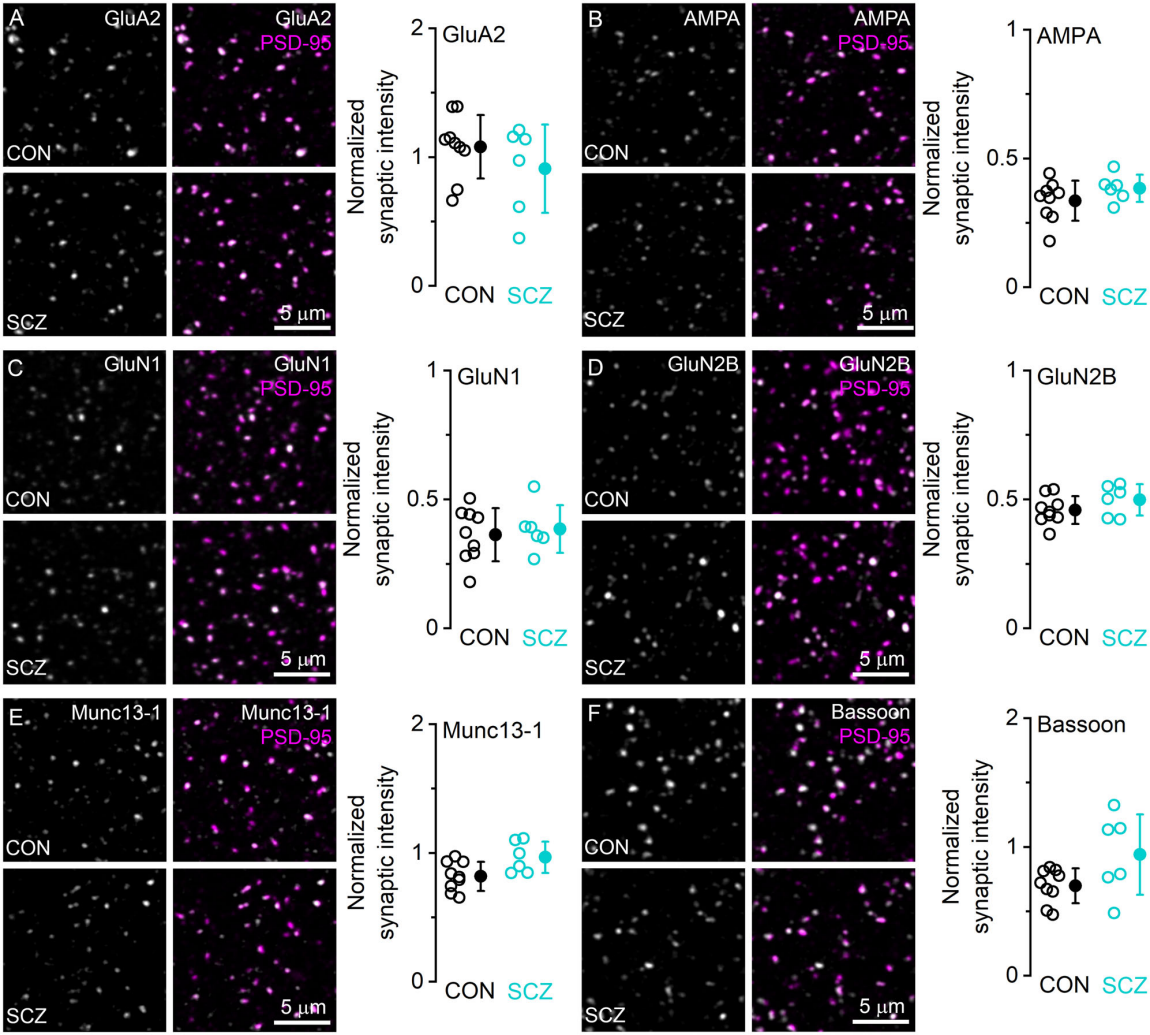
Quantitative comparison of synaptic protein content of excitatory synapses of control and schizophrenia subjects. ***A***, Colocalization of PSD-95 (magenta) and GluA2 (gray) in control (CON, top) and schizophrenia (SCZ, bottom) subjects in Layer 2 of DLPFC Area 9. ***A*** Right, Mean intensity of GluA2 immunosignal in PSD-95 immunopositive synaptic puncta in CON and SCZ subjects. GluA2 intensities were normalized to the mean PSD-95 intensity in each reaction and subject. ***B–F***, Same as in ***A***, but for all AMPA receptors (***B***) using a pan-AMPAR antibody, GluN1 (***C***), GluN2B (***D***), Munc13-1 (***E***), and Bassoon (***F***). None of the intensities differed between control and schizophrenia subjects (***A***, *p* = 0.571; ***B***, *p* = 0.821; ***C***, *p* = 0.669; ***D***, *p* = 0.621; ***E***, *p* = 0.187; ***F***, *p* = 0.285; unpaired *t* test with HBC). Open circles, mean data from individual CON (*n* = 9) and SCZ (*n* = 6) subjects; filled circles, population mean ± SD.

Synaptic glutamatergic signaling relies on postsynaptic AMPARs and NMDARs and presynaptic factors that regulate synaptic vesicle release. To reveal potential molecular alterations in glutamatergic synapses related to schizophrenia, we quantified the amount of six key pre- and postsynaptic molecules in excitatory synapses colabeled for PSD-95 in double labeling experiments (Fig. 7) in Layer 2 of DLPFC Area 9. Confocal analysis allowed us to collect a large number of synapses for each analyzed molecule from an average of 4 ± 1 reactions in both control (*n* = 9 subjects; 7,433 ± 1,708 synapses/subject) and schizophrenia (*n* = 6 subjects; 8,813 ± 2,118 synapses/subject) subjects (Table 2). Mean synaptic fluorescence for each protein was normalized to the population average of the PSD-95 signal to minimize inter-reaction variability. We first analyzed postsynaptic AMPAR and NMDAR subunits. At the population level, normalized mean GluA2 fluorescence showed a large variability among individuals and no significant difference (*p* = 0.571, unpaired *t* test with HBC) was detected between control and schizophrenia subjects (Fig. 7*A*). Similarly, the total amount of synaptic AMPARs (labeled with a pan-AMPAR antibody) was not different (*p* = 0.821; unpaired *t* test with HBC) between control and schizophrenia subjects (Fig. 7*B*). The amounts of NMDAR subunits GluN1 (Fig. 7*C*) and GluN2B (Fig. 7*D*) were also comparable between control and schizophrenia subjects (GluN1, *p* = 0.669; GluN2B, *p* = 0.621, unpaired *t* test with HBC). While there was a trend toward increased fluorescence levels of both Munc13-1 (Fig. 7*E*) and Bassoon (Fig. 7*F*) in schizophrenia subjects, multiple-comparison analysis did not reveal statistically significant differences (Munc13-1, *p* = 0.187; Bassoon, *p* = 0.285; unpaired *t* test with HBC). The trend of increased Munc13-1 immunoreactivity in schizophrenia subjects prompted us to further investigate Munc13-1 fluorescence at higher resolution.

### Quantitative STED analysis of Munc13-1 nanoclusters in the presynaptic AZ of excitatory synapses

**Figure 8.**
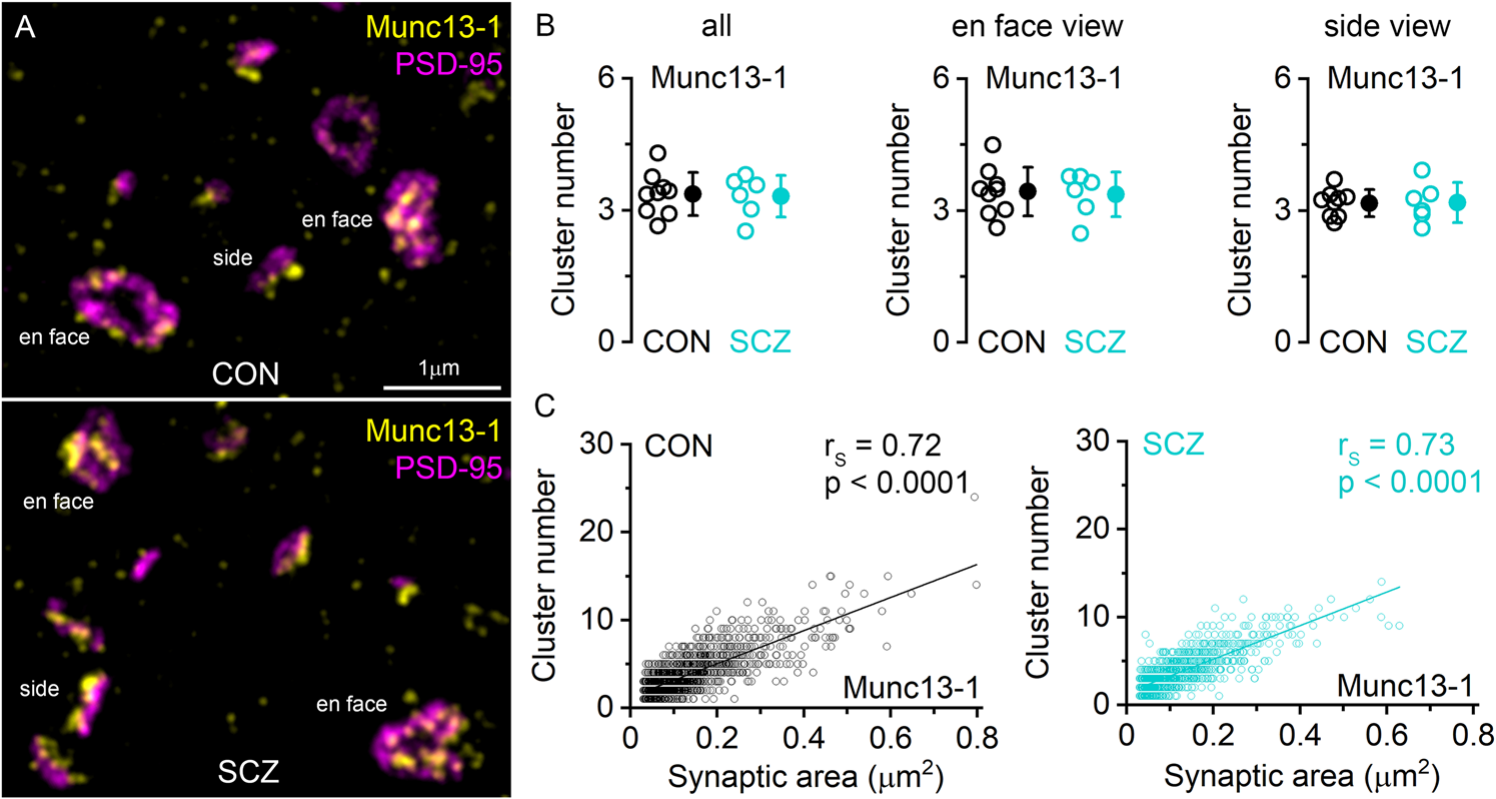
Quantitative STED analysis of Munc13-1 nanoclusters in the presynaptic AZ of excitatory synapses. ***A***, STED images of PSD-95 (magenta) and Munc13-1 (yellow) double immunofluorescent reactions in Layer 2 of DLPFC Area 9 of a control (CON, top) and a schizophrenia (SCZ, bottom) subject. Synapses with different orientations were captured, revealing both en face and side views of synapses. ***B***, The comparable number of Munc13-1 nanoclusters associated with PSD-95 immunopositive synapses in all (***B*** left, *p* = 0.861, unpaired *t* test), en face view (***B*** center, *p* = 0.831, unpaired *t* test), and side view (***B*** right, *p* = 0.951, unpaired *t* test) synapses between CON (*n* = 9 subjects) and SCZ (*n* = 6 subjects) subjects. ***C***, The number of Munc13-1 nanoclusters as a function of synaptic area in CON (left, *n* = 1,762 synapses from 9 subjects) and SCZ (right, *n* = 1,291 synapses from 6 subjects) subjects. *r*_S_, Spearman's rank correlation coefficient.

STED microscopy revealed that as in rodents, Munc13-1 fluorescence in human PSD-95–labeled excitatory synapses is also organized in subsynaptic nanoclusters within the presynaptic AZ both in control and schizophrenia subjects (Figs. 4 and 8*A*). The number of Munc13-1 nanoclusters in Layer 2 excitatory synapses was not significantly different between control and schizophrenia subjects (Table 2). Moreover, no difference was detected when en face or side view synapse orientations were analyzed separately (Fig. 8*B*). In en face synapses, the number of Munc13-1 clusters showed a similarly strong positive correlation with the synaptic size in control and schizophrenia subjects (Fig. 8*C*). This strong linear relationship between Munc13-1 clusters and the synaptic size is consistent with previous observations in mouse hippocampal excitatory synapses (Karlocai et al., 2021).

### AMPAR and NMDAR content of excitatory synapses on PV+ IN dendrites

**Figure 9.**
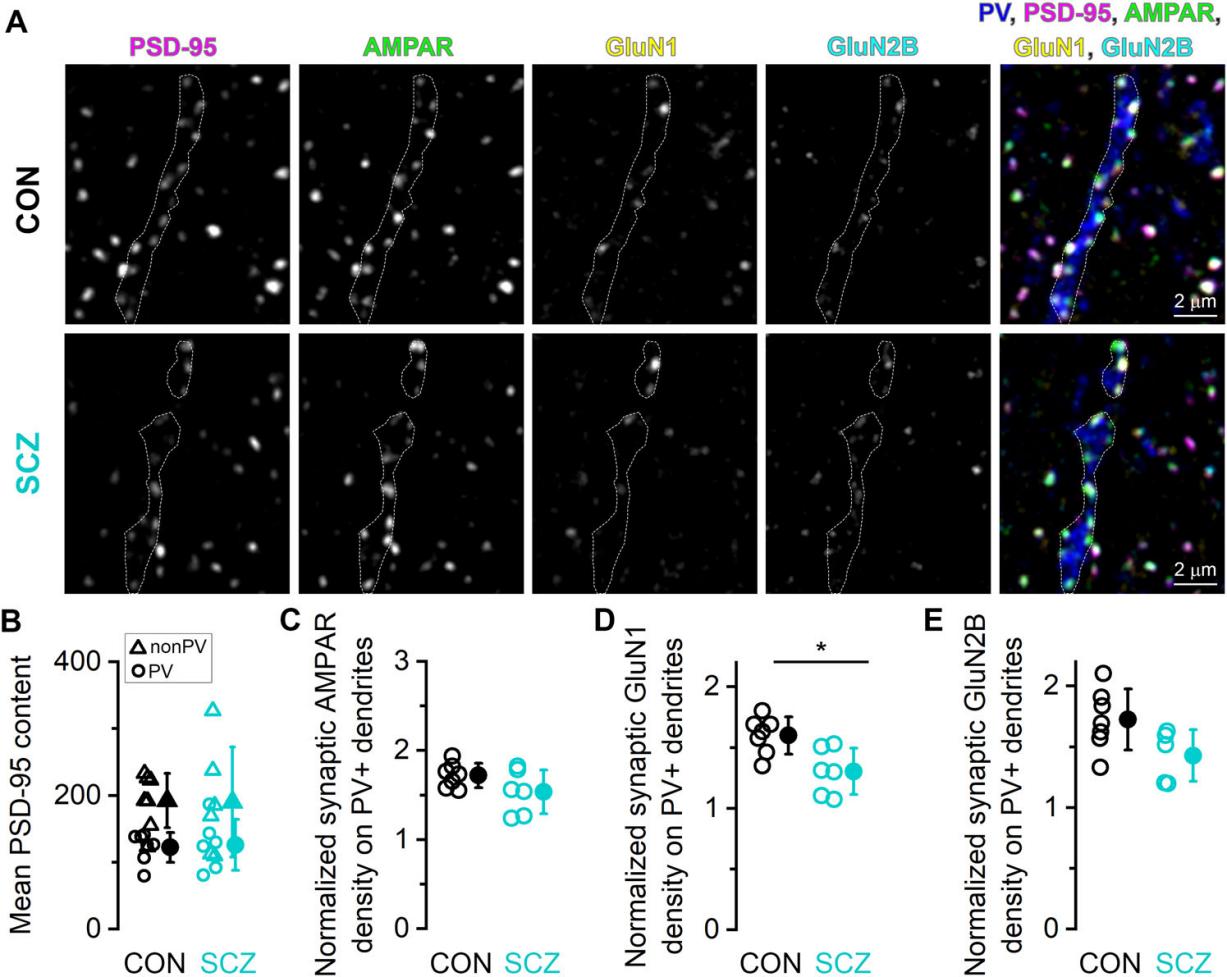
Comparison of AMPAR and NMDAR content of excitatory synapses on dendrites of PV-expressing INs of control and schizophrenia subjects. ***A***, Representative confocal images of a multiplexed immunofluorescent reaction for PV, PSD-95, AMPAR, GluN1, and GluN2B subunits taken from Layers 3/4 in the DLPFC Area 9 of a control (CON, top row) and a schizophrenia (SCZ, bottom row) subject. A PV dendrite is depicted in both subjects covered with excitatory synapses. ***B***, Mean PSD-95 integrated immunofluorescence in excitatory synapses targeting PV+ dendrites (PV) and surrounding synapses (non-PV). ***C***, Comparable AMPAR densities in excitatory synapses on PV+ dendrites in CON and SCZ subjects (*p* = 0.116, unpaired *t* test with HBC). ***D***, The mean GluN1 density in excitatory synapses on PV+ dendrites is 18% lower in SCZ compared with CON subjects (*p* = 0.031, unpaired *t* test with HBC). ***E***, Mean GluN2B densities in excitatory synapses on PV+ dendrites in SCZ compared with CON subjects (*p* = 0.117, unpaired *t* test with HBC). Densities in ***C–E*** were normalized to the population mean of the surrounding synapses. Data for ***C*** and ***D*** were collected from two experiments (CON, *n* = 7; SCZ, *n* = 6); data for ***E*** were collected from a single experiment (CON, *n* = 7, SCZ, *n* = 5). Open circles represent mean data from individual CON and SCZ subjects; filled circles indicate population mean ± SD.

While our analysis did not identify molecular alterations in upper layer DLPFC glutamatergic synapses in schizophrenia, this approach is not sensitive to potential alterations in specific subsets of excitatory synapses. Research using rodents suggests impaired glutamatergic transmission to PV+ INs might contribute to schizophrenia. Early postnatal downregulation of GluN1 expression in PV+ INs in mice produced schizophrenia-like symptoms in line with the NMDA hypofunction theory (Belforte et al., 2010; Carlen et al., 2012). We hypothesized that if schizophrenia is associated with altered glutamatergic transmission on PV+ INs, this might be reflected in alterations in the density of AMPARs and/or NMDARs at excitatory synapses targeting PV+ INs. We colabeled PSD-95, AMPARs, GluN1, GluN2B, and PV in two sequential rounds of labeling (Fig. 9*A*). PV+ dendrites were densely covered with fluorescent puncta containing PSD-95, AMPAR, and NMDARs, confirming that they are the major subcellular targets of excitatory inputs on PV+ cells (Gulyas et al., 1999; Hioki, 2015). PV+ dendrites were mainly found and sampled in Layers 3 and 4, where surrounding synapses not targeting PV+ dendrites were also collected. In these deeper layers, there was no difference in total synaptic density (*p* = 0.723, unpaired *t* test, Table 2), mean PSD-95 intensities (*p* = 0.951, unpaired *t* test), PSD-95 normalized AMPAR (*p* = 0.420, unpaired *t* test with HBC), GluN1 (*p* = 0.407, unpaired *t* test with HBC), and GluN2B (*p* = 0.245, unpaired *t* test with HBC) intensities between control and schizophrenia subjects (Fig. S2). PV+ dendrites were clearly distinguishable from thick, myelinated PV+ axons outlined by MBP (data not shown). First, we examined the density of PSD-95 immunofluorescent puncta along PV+ dendrites and found no significant difference (*p* = 0.524, unpaired *t* test) between control and schizophrenia subjects. The PSD-95 content (integrated fluorescence) of synapses on PV+ dendrites was similar in control and schizophrenia subjects (Fig. 9*B*, PV). Furthermore, they contained significantly less PSD-95 than the surrounding synapse population (Fig. 9*B*, non-PV) in both conditions (PV vs non-PV, control, *p* = 0.00024; schizophrenia, *p* = 0.035; paired *t* test). To determine the synaptic densities of AMPAR and NMDAR, we calculated AMPAR/PSD-95, GluN1/PSD-95, and GluN2B/PSD-95 ratios in each synapses targeting PV+ dendrites and normalized by the population means of surrounding synapses. AMPAR densities were larger in synapses on PV+ dendrites relative to the surrounding synapses. In line with these results, a recent immunohistochemical study in the mouse cortex also observed higher AMPAR and lower PSD-95 content in excitatory synapses on GABAergic IN dendrites relative to spine synapses (Micheva et al., 2025). We found no significant difference (*p* = 0.116, unpaired *t* test with HBC) in the normalized synaptic AMPAR density on PV+ dendrites between control and schizophrenia subjects (Fig. 9*C*). GluN1 and GluN2B receptor densities were also larger in synapses on PV+ dendrites (>1) relative to the surrounding synapses in both conditions (Fig. 9*D*,*E*). However, there was an 18% reduction (*p* = 0.031, unpaired *t* test with HBC) in GluN1 density in synapses on PV+ dendrites in schizophrenia subjects compared with that in control subjects (Fig. 9*D*). General linear regression analysis revealed no effect of age (*p* = 0.067), gender (*p* = 0.435), and PMI (*p* = 0.868) on GluN1 density. We also observed a 17% lower GluN2B density (Fig. 9*E*) in synapses on PV+ dendrites in schizophrenia subjects, which did not reach significance (*p* = 0.117, unpaired *t* test with HBC).

## Discussion

In the present study, we employed a sensitive, high-resolution multiplexed immunofluorescent localization approach to visualize and quantify key synaptic proteins in human excitatory synapses of DLPFC Area 9. To our knowledge, this is the first comprehensive demonstration of the amount and spatial organization of presynaptic molecules and glutamate receptors in individual human excitatory synapses. While we found no significant differences in synapse density, overall structure, and molecular composition, our results revealed an ∼20% reduction in the density of NMDARs in synapses targeting PV+ INs in schizophrenia subjects.

Although schizophrenia-related synaptic molecules have been extensively studied at the genetic and mRNA levels, analyzing alterations in protein levels from the postmortem tissue holds significant challenges. While immunohistochemistry offers the potential to quantitatively analyze the synapse number, size, and protein composition, it is often hampered by suboptimal fixation in the postmortem human tissue. Despite recent methodological advances in tissue preparation that improve antibody accessibility and antigenicity of target proteins in the postmortem fixed human tissue (Waldvogel, 2007; Kay et al., 2013; Woelfle et al., 2023), several proteins located in excitatory synapses remained difficult to detect. Combining epoxy resin-embedding (Holderith et al., 2020) with enhanced, microwave-assisted antigen retrieval, we successfully visualized previously undetectable proteins such as GluN1, GluN2B, and Munc13-1, among other key synaptic molecules, at individual human cortical synapses. Furthermore, multiplexed postembedding immunofluorescent reactions on ultrathin sections combined with STED superresolution microscopy enabled us to map the nanoscale spatial organization of multiple proteins within individual synapses at an unprecedented level of detail. The observed spatial arrangement of these proteins, including the clustering of Munc13-1 and GluA2, mirrored findings obtained in rodents (Masugi-Tokita and Shigemoto, 2007; Tang et al., 2016; Szoboszlay et al., 2017; Sakamoto et al., 2018; Aldahabi et al., 2022). Notably, PSD-95 immunolabeling was rather uniform within synapses, which remain stable across multiple rounds of elution/restaining, underscoring the robustness of our approach, ensuring minimal disruption of synaptic architecture.

### Preserved synapse size and density in Layer 2 of DLPFC in schizophrenia subjects

The role of excitatory synaptic dysfunction in the pathophysiology of schizophrenia is strongly supported by extensive research. Previously, alterations in synaptic density were studied using various techniques, focusing on spine and axon terminal counts. Golgi impregnation and spinophilin immunoreactions revealed reduced spine density in Layer 3 PC dendrites within prefrontal, temporal, and auditory cortices, but not in more superficial layers (Garey et al., 1998; Sweet et al., 2009; MacDonald et al., 2017), or Layers 5 and 6 (Kolluri et al., 2005). Conflicting data have been published for mRNA and protein levels of synaptophysin, a protein that is widely used as a presynaptic marker (Hu et al., 2015; Osimo et al., 2019). However, quantitative examination of immunoreactive puncta within the auditory cortex demonstrated that the number of puncta for synaptophysin or vesicular glutamate transporters VGLUT1 and VGLUT2 was minimally altered or remained unchanged in schizophrenia (Sweet et al., 2007; Moyer et al., 2013). On the postsynaptic side, PSD-95, a crucial postsynaptic scaffolding protein for glutamate receptors, also showed regional variations in mRNA transcript and protein levels, yet no schizophrenia-associated alterations were observed in the DLPFC (Kristiansen et al., 2006; Osimo et al., 2019). Given the specific expression of PSD-95 in excitatory synapses and its linear correlation with the synaptic size (Karlocai et al., 2021; Micheva et al., 2025), it is ideally suited for immunohistochemical identification and characterization of individual excitatory synapses in postmortem tissue. Our findings revealed no significant differences in synapse density, synaptic area, and PSD-95 intensity between schizophrenia and control subjects. STED microscopy confirmed these results, showing the comparable synapse size and PSD-95 intensity and a consistent correlation between PSD-95 fluorescence and synapse size across both subject groups. Our results suggest that synaptic density and excitatory synapse size in upper layers of DLPFC are preserved in schizophrenia, challenging the concept of widespread synaptic deficits in this condition.

### Comparable levels of synaptic glutamate receptors in control and schizophrenia subjects

Glutamatergic transmission critically depends on AMPAR and NMDAR as well as dozens of key presynaptic proteins, essential for localized, precisely timed neurotransmitter release. Previous studies on glutamate receptor mRNA levels in schizophrenia have yielded conflicting results, failing to establish clear patterns of change between diseased and control brain tissues (Hu et al., 2015). This inconsistency is evident in the prefrontal cortex, where reduced, unaltered, or increased levels of mRNA have been reported for NMDAR and AMPAR subunit (Akbarian et al., 1996; Sokolov, 1998; Dracheva et al., 2001; O'Connor and Hemby, 2007; Beneyto and Meador-Woodruff, 2008; Weickert et al., 2013). Studies investigating glutamate receptor proteins in the same frontal region also show contradictory results (Kristiansen et al., 2006; Corti et al., 2011; Weickert et al., 2013). These analyses lack the necessary spatial resolution to distinguish between layer- or cell type-specific differences in synaptic protein densities and distribution at individual synapses. Using postembedding immunohistochemistry, we explored the abundance of key synaptic proteins in the DLPFC of schizophrenia patients. Surprisingly, postsynaptic AMPA (GluA2 and pan-AMPAR) and NMDA (GluN1, GluN2B) receptor levels showed no significant differences between control and schizophrenia subjects. Our findings confirm some previous studies, using different methodologies, demonstrating the lack of gross alterations in synaptic glutamate receptor levels.

### The number of neurotransmitter release sites is unchanged in schizophrenia subjects

The strength of a synapse is determined by the vesicle release probability, quantal size, and the number of vesicle release sites. These parameters can be directly determined using functional approaches only, such as paired whole-cell recordings and quantal analysis of the postsynaptic responses. However, they can be indirectly estimated from the molecular constituents of the synapses and nanotopology of key pre- and postsynaptic molecules within the AZ/PSD. For example, the quantal size is primarily determined by the density of AMPARs in the PSD, whereas the number of release sites strongly correlates with the number of Munc13-1 nanoclusters within the AZs (Reddy-Alla et al., 2017; Sakamoto et al., 2018; Karlocai et al., 2021) and therefore can be used as molecular markers for vesicular release sites (Karlocai, 2021; Aldahabi, 2022). In rodent excitatory synapses, the number functional release sites and the number of Munc13-1 nanoclusters (Karlocai et al., 2021; Aldahabi et al., 2022) scale linearly with the size of the AZ. Consistent with our studies in rodents, we observed that Munc13-1 formed nanoclusters within the presynaptic AZ, the number of which scaled linear with the size of human excitatory synapses, demonstrating similar intricate spatial organization. The lack of significant differences in AMPAR density and Munc13-1 nanocluster number between schizophrenia and control groups indicates that the quantal size and the number of release sites are likely to be similar in cortical excitatory synapses of control and schizophrenia subjects. Nonetheless, future studies are needed to determine potential schizophrenia-related changes of other key presynaptic molecules, protein–protein interactions (Ramos-Miguel et al., 2015), and the molecular nanotopology of vesicle release sites and voltage-gated Ca^2+^ channels (Rebola et al., 2019), which would indicate potential changes in neurotransmitter release probability.

### Altered NMDAR content of excitatory synapses on PV+ INs in schizophrenia subjects

Although our population-level analysis provided an overview of excitatory synapses in upper layers of the DLPFC, it incorporated synapses on different types of nerve cells. To gain deeper insights, we moved beyond the population-level analysis and specifically examined excitatory synapses targeting PV+ INs, an IN type that has been directly implicated in schizophrenia. We determined the NMDAR and AMPAR content of excitatory synapses on PV+ dendrites. Our choice of selection was motivated by previous studies demonstrating that interfering with NMDAR expression selectively in PV+ INs (Belforte et al., 2010; Korotkova et al., 2010; Carlen et al., 2012) is accompanied by deficits in cortical gamma oscillations and cognitive impairment reminiscent to schizophrenia. While synapse density, PSD-95, and AMPAR content of synapses were comparable between groups, we observed a significant reduction in GluN1 density and a trend toward reduced GluN2B density at synapses innervating PV+ dendrites in schizophrenia subjects. This slight reduction of NMDAR content may contribute to the lower in vivo activity of PV+ cells. This finding is consistent with the NMDA hypofunction theory and suggests that alterations in glutamatergic transmission onto PV+ INs might cause disinhibition of PCs that eventually leads to hyperexcitability, disrupted gamma oscillations, and cognitive deficits observed in schizophrenia. The roles of GluN2A and GluN2D subunits remain to be determined when suitable antibodies will be available for investigations in the human tissue.

In conclusion, our study provides valuable insights into the molecular architecture of excitatory synapses in the DLPFC of control and schizophrenia subjects. While we found no significant differences in the overall density, size, and protein content of excitatory synapses in upper layers, we observed a significant reduction in NMDAR density at synapses innervating PV+ IN dendrites. This finding suggests that the pathophysiology of schizophrenia involves subtle, circuit-specific alterations in synaptic function rather than gross changes in overall synaptic abundance/strength. Our results highlight the importance of investigating specific subsets of synapses and employing high-resolution techniques to unravel the complex molecular underpinnings of this debilitating disorder.

## References

[B1] Akbarian S, Sucher NJ, Bradley D, Tafazzoli A, Trinh D, Hetrick WP, Potkin SG, Sandman CA, Bunney WE Jr, Jones EG (1996) Selective alterations in gene expression for NMDA receptor subunits in prefrontal cortex of schizophrenics. J Neurosci 16:19–30. 10.1523/JNEUROSCI.16-01-00019.1996 8613785 PMC6578738

[B2] Aldahabi M, Balint F, Holderith N, Lorincz A, Reva M, Nusser Z (2022) Different priming states of synaptic vesicles underlie distinct release probabilities at hippocampal excitatory synapses. Neuron 110:4144–4161.e7. 10.1016/j.neuron.2022.09.035 36261033 PMC9796815

[B3] Atwood HL, Karunanithi S (2002) Diversification of synaptic strength: presynaptic elements. Nat Rev 3:497–516. 10.1038/nrn876 12094207

[B4] Augustin I, Rosenmund C, Sudhof TC, Brose N (1999) Munc13-1 is essential for fusion competence of glutamatergic synaptic vesicles. Nature 400:457–461. 10.1038/22768 10440375

[B5] Batiuk MY, et al. (2022) Upper cortical layer-driven network impairment in schizophrenia. Sci Adv 8:eabn8367. 10.1126/sciadv.abn8367 36223459 PMC9555788

[B6] Bayes A, Grant SG (2009) Neuroproteomics: understanding the molecular organization and complexity of the brain. Nat Rev 10:635–646. 10.1038/nrn2701 19693028

[B8] Belforte JE, Zsiros V, Sklar ER, Jiang Z, Yu G, Li Y, Quinlan EM, Nakazawa K (2010) Postnatal NMDA receptor ablation in corticolimbic interneurons confers schizophrenia-like phenotypes. Nat Neurosci 13:76–83. 10.1038/nn.2447 19915563 PMC2797836

[B9] Beneyto M, Meador-Woodruff JH (2008) Lamina-specific abnormalities of NMDA receptor-associated postsynaptic protein transcripts in the prefrontal cortex in schizophrenia and bipolar disorder. Neuropsychopharmacology 33:2175–2186. 10.1038/sj.npp.1301604 18033238

[B10] Brose N, Hofmann K, Hata Y, Sudhof TC (1995) Mammalian homologues of *Caenorhabditis elegans* unc-13 gene define novel family of C2-domain proteins. J Biol Chem 270:25273–25280. 10.1074/jbc.270.42.25273 7559667

[B11] Callicott JH, Mattay VS, Verchinski BA, Marenco S, Egan MF, Weinberger DR (2003) Complexity of prefrontal cortical dysfunction in schizophrenia: more than up or down. Am J Psychiatry 160:2209–2215. 10.1176/appi.ajp.160.12.2209 14638592

[B12] Carlen M, et al. (2012) A critical role for NMDA receptors in parvalbumin interneurons for gamma rhythm induction and behavior. Mol Psychiatry 17:537–548. 10.1038/mp.2011.31 21468034 PMC3335079

[B13] Catts VS, Derminio DS, Hahn CG, Weickert CS (2015) Postsynaptic density levels of the NMDA receptor NR1 subunit and PSD-95 protein in prefrontal cortex from people with schizophrenia. NPJ Schizophr 1:15037. 10.1038/npjschz.2015.37 27336043 PMC4849460

[B14] Chen CH, Huang YS, Liao DL, Huang CY, Lin CH, Fang TH (2021) Identification of rare mutations of two presynaptic cytomatrix genes BSN and PCLO in schizophrenia and bipolar disorder. J Pers Med 11:1057. 10.3390/jpm11111057 34834409 PMC8625612

[B15] Cho RY, Konecky RO, Carter CS (2006) Impairments in frontal cortical gamma synchrony and cognitive control in schizophrenia. Proc Natl Acad Sci U S A 103:19878–19883. 10.1073/pnas.0609440103 17170134 PMC1750867

[B16] Chung DW, Fish KN, Lewis DA (2016) Pathological basis for deficient excitatory drive to cortical parvalbumin interneurons in schizophrenia. Am J Psychiatry 173:1131–1139. 10.1176/appi.ajp.2016.16010025 27444795 PMC5089927

[B17] Conti F, Minelli A, Molnar M, Brecha NC (1994) Cellular localization and laminar distribution of NMDAR1 mRNA in the rat cerebral cortex. J Comp Neurol 343:554–565. 10.1002/cne.903430406 8034787

[B18] Corti C, Xuereb JH, Crepaldi L, Corsi M, Michielin F, Ferraguti F (2011) Altered levels of glutamatergic receptors and Na+/K+ ATPase-alpha1 in the prefrontal cortex of subjects with schizophrenia. Schizophr Res 128:7–14. 10.1016/j.schres.2011.01.021 21353485

[B19] Dracheva S, Marras SA, Elhakem SL, Kramer FR, Davis KL, Haroutunian V (2001) N-methyl-D-aspartic acid receptor expression in the dorsolateral prefrontal cortex of elderly patients with schizophrenia. Am J Psychiatry 158:1400–1410. 10.1176/appi.ajp.158.9.1400 11532724

[B20] Enwright JF, Sanapala S, Foglio A, Berry R, Fish KN, Lewis DA (2016) Reduced labeling of parvalbumin neurons and perineuronal nets in the dorsolateral prefrontal cortex of subjects with schizophrenia. Neuropsychopharmacology 41:2206–2214. 10.1038/npp.2016.24 26868058 PMC4946056

[B21] Fritschy J-M, Weinmann O, Wenzel A, Benke D (1998) Synapse-specific localization of NMDA and GABA_A_ receptor subunits revealed by antigen-retrieval immunohistochemistry. J Comp Neurol 390:194–210. 10.1002/(SICI)1096-9861(19980112)390:2<194::AID-CNE3>3.0.CO;2-X 9453664

[B22] Fromer M, et al. (2014) De novo mutations in schizophrenia implicate synaptic networks. Nature 506:179–184. 10.1038/nature12929 24463507 PMC4237002

[B23] Fung SJ, Webster MJ, Sivagnanasundaram S, Duncan C, Elashoff M, Weickert CS (2010) Expression of interneuron markers in the dorsolateral prefrontal cortex of the developing human and in schizophrenia. Am J Psychiatry 167:1479–1488. 10.1176/appi.ajp.2010.09060784 21041246

[B24] Garey LJ, Ong WY, Patel TS, Kanani M, Davis A, Mortimer AM, Barnes TR, Hirsch SR (1998) Reduced dendritic spine density on cerebral cortical pyramidal neurons in schizophrenia. J Neurol Neurosurg Psychiatry 65:446–453. 10.1136/jnnp.65.4.446 9771764 PMC2170311

[B25] Glausier JR, Fish KN, Lewis DA (2014) Altered parvalbumin basket cell inputs in the dorsolateral prefrontal cortex of schizophrenia subjects. Mol Psychiatry 19:30–36. 10.1038/mp.2013.152 24217255 PMC3874728

[B26] Gulyas AI, Megias M, Emri Z, Freund TF (1999) Total number and ratio of excitatory and inhibitory synapses converging onto single interneurons of different types in the CA1 area of the rat hippocampus. J Neurosci 19:10082–10097. 10.1523/JNEUROSCI.19-22-10082.1999 10559416 PMC6782984

[B27] Haenschel C, Bittner RA, Waltz J, Haertling F, Wibral M, Singer W, Linden DE, Rodriguez E (2009) Cortical oscillatory activity is critical for working memory as revealed by deficits in early-onset schizophrenia. J Neurosci 29:9481–9489. 10.1523/JNEUROSCI.1428-09.2009 19641111 PMC6666530

[B28] Hashimoto T, Volk DW, Eggan SM, Mirnics K, Pierri JN, Sun Z, Sampson AR, Lewis DA (2003) Gene expression deficits in a subclass of GABA neurons in the prefrontal cortex of subjects with schizophrenia. J Neurosci 23:6315–6326. 10.1523/JNEUROSCI.23-15-06315.2003 12867516 PMC6740534

[B29] Hioki H (2015) Compartmental organization of synaptic inputs to parvalbumin-expressing GABAergic neurons in mouse primary somatosensory cortex. Anat Sci Int 90:7–21. 10.1007/s12565-014-0264-8 25467527

[B30] Holderith N, Heredi J, Kis V, Nusser Z (2020) A high-resolution method for quantitative molecular analysis of functionally characterized individual synapses. Cell Rep 32:107968. 10.1016/j.celrep.2020.107968 32726631 PMC7408500

[B31] Hu W, MacDonald ML, Elswick DE, Sweet RA (2015) The glutamate hypothesis of schizophrenia: evidence from human brain tissue studies. Ann N Y Acad Sci 1338:38–57. 10.1111/nyas.12547 25315318 PMC4363164

[B32] Karlocai MR, Heredi J, Benedek T, Holderith N, Lorincz A, Nusser Z (2021) Variability in the Munc13-1 content of excitatory release sites. Elife 10:e67468. 10.7554/eLife.67468 33904397 PMC8116053

[B33] Kay KR, et al. (2013) Studying synapses in human brain with array tomography and electron microscopy. Nat Protoc 8:1366–1380. 10.1038/nprot.2013.078 23787894 PMC3712649

[B34] Kirov G, et al. (2012) De novo CNV analysis implicates specific abnormalities of postsynaptic signalling complexes in the pathogenesis of schizophrenia. Mol Psychiatry 17:142–153. 10.1038/mp.2011.154 22083728 PMC3603134

[B35] Kolluri N, Sun Z, Sampson AR, Lewis DA (2005) Lamina-specific reductions in dendritic spine density in the prefrontal cortex of subjects with schizophrenia. Am J Psychiatry 162:1200–1202. 10.1176/appi.ajp.162.6.1200 15930070

[B36] Konno K, Yamasaki M, Miyazaki T, Watanabe M (2023) Glyoxal fixation: an approach to solve immunohistochemical problem in neuroscience research. Sci Adv 9:eadf7084. 10.1126/sciadv.adf7084 37450597 PMC10348680

[B37] Korotkova T, Fuchs EC, Ponomarenko A, von Engelhardt J, Monyer H (2010) NMDA receptor ablation on parvalbumin-positive interneurons impairs hippocampal synchrony, spatial representations, and working memory. Neuron 68:557–569. 10.1016/j.neuron.2010.09.017 21040854

[B38] Kristiansen LV, Beneyto M, Haroutunian V, Meador-Woodruff JH (2006) Changes in NMDA receptor subunits and interacting PSD proteins in dorsolateral prefrontal and anterior cingulate cortex indicate abnormal regional expression in schizophrenia. Mol Psychiatry 11:737–747. 705. 10.1038/sj.mp.4001844 16702973

[B39] Krystal JH, Karper LP, Seibyl JP, Freeman GK, Delaney R, Bremner JD, Heninger GR, Bowers MB Jr, Charney DS (1994) Subanesthetic effects of the noncompetitive NMDA antagonist, ketamine, in humans. Psychotomimetic, perceptual, cognitive, and neuroendocrine responses. Arch Gen Psychiatry 51:199–214. 10.1001/archpsyc.1994.03950030035004 8122957

[B40] Lewis DA, Mirnics K (2006) Transcriptome alterations in schizophrenia: disturbing the functional architecture of the dorsolateral prefrontal cortex. Prog Brain Res 158:141–152. 10.1016/S0079-6123(06)58007-0 17027695

[B41] Lipstein N, et al. (2017) Synaptic UNC13A protein variant causes increased neurotransmission and dyskinetic movement disorder. J Clin Invest 127:1005–1018. 10.1172/JCI90259 28192369 PMC5330740

[B42] Lorincz A, Nusser Z (2010) Molecular identity of dendritic voltage-gated sodium channels. Science 328:906–909. 10.1126/science.1187958 20466935 PMC3546315

[B43] MacDonald ML, et al. (2017) Selective loss of smaller spines in schizophrenia. Am J Psychiatry 174:586–594. 10.1176/appi.ajp.2017.16070814 28359200 PMC5800878

[B44] Masugi-Tokita M, Tarusawa E, Watanabe M, Molnar E, Fujimoto K, Shigemoto R (2007) Number and density of AMPA receptors in individual synapses in the rat cerebellum as revealed by SDS-digested freeze-fracture replica labeling. J Neurosci 27:2135–2144. 10.1523/JNEUROSCI.2861-06.2007 17314308 PMC6673557

[B45] Masugi-Tokita M, Shigemoto R (2007) High-resolution quantitative visualization of glutamate and GABA receptors at central synapses. Curr Opin Neurobiol 17:387–393. 10.1016/j.conb.2007.04.012 17499496

[B46] Micheva KD, Simhal AK, Schardt J, Smith SJ, Weinberg RJ, Owen SF (2025) Data-driven synapse classification reveals a logic of glutamate receptor diversity. bioRxiv.

[B47] Millan MJ, et al. (2016) Altering the course of schizophrenia: progress and perspectives. Nat Rev Drug Discov 15:485–515. 10.1038/nrd.2016.28 26939910

[B48] Montenegro-Venegas C, Guhathakurta D, Pina-Fernandez E, Andres-Alonso M, Plattner F, Gundelfinger ED, Fejtova A (2022) Bassoon controls synaptic vesicle release via regulation of presynaptic phosphorylation and cAMP. EMBO Rep 23:e53659. 10.15252/embr.202153659 35766170 PMC9346490

[B49] Moyer CE, Delevich KM, Fish KN, Asafu-Adjei JK, Sampson AR, Dorph-Petersen KA, Lewis DA, Sweet RA (2013) Intracortical excitatory and thalamocortical boutons are intact in primary auditory cortex in schizophrenia. Schizophr Res 149:127–134. 10.1016/j.schres.2013.06.024 23830684 PMC3756893

[B50] O'Connor JA, Hemby SE (2007) Elevated GRIA1 mRNA expression in layer II/III and V pyramidal cells of the DLPFC in schizophrenia. Schizophr Res 97:277–288. 10.1016/j.schres.2007.09.022 17942280 PMC3255089

[B51] Osimo EF, Beck K, Reis Marques T, Howes OD (2019) Synaptic loss in schizophrenia: a meta-analysis and systematic review of synaptic protein and mRNA measures. Mol Psychiatry 24:549–561. 10.1038/s41380-018-0041-5 29511299 PMC6004314

[B52] Purcell SM, et al. (2014) A polygenic burden of rare disruptive mutations in schizophrenia. Nature 506:185–190. 10.1038/nature12975 24463508 PMC4136494

[B53] Ramos-Miguel A, Beasley CL, Dwork AJ, Mann JJ, Rosoklija G, Barr AM, Honer WG (2015) Increased SNARE protein-protein interactions in orbitofrontal and anterior cingulate cortices in schizophrenia. Biol Psychiatry 78:361–373. 10.1016/j.biopsych.2014.12.012 25662103 PMC4474796

[B54] Rebola N, Reva M, Kirizs T, Szoboszlay M, Lorincz A, Moneron G, Nusser Z, DiGregorio DA (2019) Distinct nanoscale calcium channel and synaptic vesicle topographies contribute to the diversity of synaptic function. Neuron 104:693–710.e9. 10.1016/j.neuron.2019.08.014 31558350

[B55] Reddy-Alla S, et al. (2017) Stable positioning of Unc13 restricts synaptic vesicle fusion to defined release sites to promote synchronous neurotransmission. Neuron 95:1350–1364.e12. 10.1016/j.neuron.2017.08.016 28867551

[B56] Rubio MD, Drummond JB, Meador-Woodruff JH (2012) Glutamate receptor abnormalities in schizophrenia: implications for innovative treatments. Biomol Ther 20:1–18. 10.4062/biomolther.2012.20.1.001 PMC3792192 24116269

[B57] Ruzicka WB, et al. (2024) Single-cell multi-cohort dissection of the schizophrenia transcriptome. Science 384:eadg5136. 10.1126/science.adg5136 38781388 PMC12772489

[B58] Sakamoto H, Ariyoshi T, Kimpara N, Sugao K, Taiko I, Takikawa K, Asanuma D, Namiki S, Hirose K (2018) Synaptic weight set by Munc13-1 supramolecular assemblies. Nat Neurosci 21:41–49. 10.1038/s41593-017-0041-9 29230050

[B59] Sigurdsson T, Duvarci S (2015) Hippocampal-prefrontal interactions in cognition, behavior and psychiatric disease. Front Syst Neurosci 9:190. 10.3389/fnsys.2015.00190 26858612 PMC4727104

[B60] Sokolov BP (1998) Expression of NMDAR1, GluR1, GluR7, and KA1 glutamate receptor mRNAs is decreased in frontal cortex of “neuroleptic-free” schizophrenics: evidence on reversible up-regulation by typical neuroleptics. J Neurochem 71:2454–2464. 10.1046/j.1471-4159.1998.71062454.x 9832144

[B61] Sudhof TC (2012) The presynaptic active zone. Neuron 75:11–25. 10.1016/j.neuron.2012.06.012 22794257 PMC3743085

[B62] Sweet RA, Bergen SE, Sun Z, Marcsisin MJ, Sampson AR, Lewis DA (2007) Anatomical evidence of impaired feedforward auditory processing in schizophrenia. Biol Psychiatry 61:854–864. 10.1016/j.biopsych.2006.07.033 17123477

[B63] Sweet RA, Henteleff RA, Zhang W, Sampson AR, Lewis DA (2009) Reduced dendritic spine density in auditory cortex of subjects with schizophrenia. Neuropsychopharmacology 34:374–389. 10.1038/npp.2008.67 18463626 PMC2775717

[B64] Szoboszlay M, Kirizs T, Nusser Z (2017) Objective quantification of nanoscale protein distributions. Sci Rep 7:15240. 10.1038/s41598-017-15695-w 29127366 PMC5681686

[B65] Tang AH, Chen H, Li TP, Metzbower SR, MacGillavry HD, Blanpied TA (2016) A trans-synaptic nanocolumn aligns neurotransmitter release to receptors. Nature 536:210–214. 10.1038/nature19058 27462810 PMC5002394

[B66] Trubetskoy V, et al. (2022) Mapping genomic loci implicates genes and synaptic biology in schizophrenia. Nature 604:502–508. 10.1038/s41586-022-04434-5 35396580 PMC9392466

[B67] Uhlhaas PJ, Singer W (2015) Oscillations and neuronal dynamics in schizophrenia: the search for basic symptoms and translational opportunities. Biol Psychiatry 77:1001–1009. 10.1016/j.biopsych.2014.11.019 25676489

[B68] Volk DW, Matsubara T, Li S, Sengupta EJ, Georgiev D, Minabe Y, Sampson A, Hashimoto T, Lewis DA (2012) Deficits in transcriptional regulators of cortical parvalbumin neurons in schizophrenia. Am J Psychiatry 169:1082–1091. 10.1176/appi.ajp.2012.12030305 22983435 PMC3513625

[B69] Waldvogel FA (2007) Ultrasound–now also for microbiologists? N Engl J Med 357:705–706. 10.1056/NEJMe078047 17699821

[B70] Watanabe M, Fukaya M, Sakimura K, Manabe T, Mishina M, Inoue Y (1998) Selective scarcity of NMDA receptor channel subunits in the stratum lucidum (mossy fibre-recipient layer) of the mouse hippocampal CA3 subfield. Eur J Neurosci 10:478–487. 10.1046/j.1460-9568.1998.00063.x 9749710

[B71] Weickert CS, et al. (2013) Molecular evidence of N-methyl-D-aspartate receptor hypofunction in schizophrenia. Mol Psychiatry 18:1185–1192. 10.1038/mp.2012.137 23070074 PMC3807670

[B72] Woelfle S, Deshpande D, Feldengut S, Braak H, Del Tredici K, Roselli F, Deisseroth K, Michaelis J, Boeckers TM, Schon M (2023) CLARITY increases sensitivity and specificity of fluorescence immunostaining in long-term archived human brain tissue. BMC Biol 21:113. 10.1186/s12915-023-01582-6 37221592 PMC10207789

